# The emotional effect of terrorism

**DOI:** 10.1038/s41598-024-77350-5

**Published:** 2024-11-03

**Authors:** Vincenzo Bove, Georgios Efthyvoulou, Armine Ghazaryan, Harry Pickard

**Affiliations:** 1 IMT School for Advanced Studies Lucca, Lucca, 55100 Italy; 2 School of Economics, University of Sheffield, Sheffield, S1 4DT UK; 3 Department of Economics, University of Southampton, Southampton, SO17 1BJ UK; 4 Newcastle University Business School, Newcastle University, Newcastle, NE1 4SE UK

**Keywords:** Terrorism, Sentiments, Emotions, Twitter, Risk factors, Statistics

## Abstract

Terrorism causes emotional reactions among public audiences, with downstream consequences for their well-being, attitudes and policy preferences. We utilise a novel approach which harnesses a unique dataset of Twitter activity from 324K users to precisely capture emotional responses to terrorism. Our results demonstrate that terrorist attacks induce dramatic spikes in various discrete emotions of a negative valence, which vary based on the characteristics of the attacks. Furthermore, we show that the terrorism-induced effects on emotions are accompanied by changes in feelings about immigration.

## Introduction

The impact of terrorism reaches far beyond its immediate victims. As a form of ‘psychological warfare’^1^ or a tactic designed to ‘precipitate political change’^2^, terrorism aims to instil feelings of fear, anxiety, and insecurity. As such, it is only successful insofar as it triggers emotional reactions and engenders changes in public attitudes. At the same time, as a social category referring to acts of illegitimate extreme violence, terrorism provokes particular discursive reactions within certain cultural-political contexts^3^. The concept of terrorism is not only articulated by state elites and through ‘hard’ security policy, but also at the level of the ‘everyday’ through vernacular practices^4^. From this perspective, it can only be properly analysed by considering the dynamics of the public reactions and intersubjective judgements it provokes. Evaluating how terrorist acts influence public emotions, cognitions, and discourses allows us to measure the indirect (non-economic) costs of terrorism, and to assess the effectiveness of terrorist tactics in achieving their political and social goals^5,6^.

Our emotional experiences play an important role in our long-term subjective well-being, flourishing, and life satisfaction^7,8^. Terrorism, by its very nature, is intended to elicit strong emotional reactions, which can have detrimental effects on mental health^9,10^ and increase trauma- and stressor-related disorders, both within and outside the target country^11,12^. Our emotions also shape our cognition, processing, decision-making and normative judgments^13–17^. Notably, exposure to terrorism can alter the public’s cognition and policy preferences even when the immediate emotional reactions are short-lived^18–22^. Disaggregating the consequences of attacks based on discrete emotional states among audiences has broader implications for attitudinal responses, as divergent emotional reactions can provoke distinct policy preferences^20,21,23^, often resulting in the inauguration of harsh policy responses^24,25^. The pervasive influence of some specific negative emotions in contemporary politics yields profound consequences, as shown by recent studies on the intersection of emotion and politics^26^. For example, anger tends to bolster support for vindictive policies while diminishing endorsement for conciliatory measures. Conversely, fear leads to stronger support for precautionary defense measures in the presence of external threats such as terrorism^26,27^. Fear and anxiety are also associated with individuals’ inclination towards adopting right-wing populist attitudes^26,28^.

The advent of social media has accelerated and intensified the dissemination of information^29^, making the effects of terrorism even more far-reaching and problematic than before. Social media is viewed as a particularly productive object of study because it is an arena which hosts ‘everyday’ security speak and provides a dynamic view of how violent events are processed by the public in real time^30^. Social media acts as a critical site at which the ‘trauma’ of terrorist incidents is processed, and novel security discourses emerge^31^. Twitter reactions, in particular, host evolving political narratives about the causes of terrorism and state security policy^4^.

Against this background, our study explores the emotional effect of eight major terrorist incidents that occurred in the United Kingdom (UK) between 2016 and 2020, using a large and unique dataset encompassing 7.6 million observations collected from 324K Twitter users. Though there has been some exploration of utilising Twitter to understand reactions to terrorism^32,33^, no previous studies have employed such a large dataset from a single country covering multiple and heterogeneous attacks, with different severity levels and perpetrator types. Furthermore, none of the existing Twitter-based studies have focused on the first-order effects of terrorism; that is, the terrorism-induced changes in people’s overall emotional state.

The use of Twitter data offers a unique opportunity to gain insight into public reactions to terrorist acts. Users of Twitter can comment news and communicate their views on current events from their own accounts in *real time*^33^. As such, contrary to questionnaires, analysing Twitter data allows us to track changes in an individual’s attitudes and emotions within very short time intervals – e.g., every few minutes or hours – which can help to produce valid causal estimates of theoretically relevant shocks. In addition, it allows us to study a person’s emotions and attitudes based on their own language and frames of reference, rather than their responses to survey questions, which are inherently subject to some misinterpretation and bias. Although we cannot infer to the whole UK population more broadly, focusing on Twitter users allows us to perform a comparatively hard test of the terrorism-induced effects on emotions and attitudes, as these outcomes should be generally more stable among social media users who are exposed to multiple news stories at the same time^34^.

To isolate the causal effect of terrorist attacks on emotions, we focus on a short time window (from 3 days before up to and including 3 days after each attack) and exploit variation within individuals, net of potential temporal unobserved factors. In this way, we can estimate whether the tweets posted by a given individual in the short period after a specific attack convey more negative feelings than those posted by the same individual in the short period before the same attack. To lend further credibility to our causal claims, we also report an array of different specifications and robustness tests.

Our analysis reveals that terrorist attacks induce dramatic spikes in various discrete emotions of a negative valence, and that fear and anger are the emotions that display the largest and most persistent post-attack rise. This is consistent with extant research suggesting that fear and anger, rather than other negative emotions such as sadness, are the dominant reactions triggered by terrorist incidents^22^. Violent acts prime a particular set of cognitive responses linked to these emotions, including thoughts about the inevitability of death^35–37^, the idea that one’s country is in danger^38^, and the perceptions of injustice^39^. More precisely, fear stems from an appraisal of heightened personal risk or weakness, whereas anger is triggered by appraisals of comparative strength, combined with a desire for vengeance against perpetrators^40^.

The observed patterns persist when we focus on non-terror-related tweets, suggesting that terrorism is having the intended effect of upsetting and distressing people in general beyond the act itself. Finally, we find that the terrorism-induced effects on emotions are accompanied by heightened negative feelings about immigration. To the extent that social media emotional trends can predict changes in offline behaviour and real-life outcomes^41–43^, our findings can speak to the broader societal ramifications of terrorist attacks.

## Results

### The emotional effect of terrorism

We start by comparing the emotional content of tweets posted 3 days after an attack to that of tweets posted 3 days before the attack. Panel A of Table 1 reports the post-attack change in the overall negative sentiment (column 1) and in four negative emotions: fear, anger, sadness, and disgust (columns 2–5). We can see that all emotions of negative valence are heightened in the aftermath of terrorist attacks: the treatment (post-attack) effect is positive and highly statistically significant throughout. Comparing the estimates in the last four columns, we can also see that fear and anger are the emotions that display that largest increase. Importantly, these baseline results remain essentially the same when we add the fixed effects and control variables in a progressive manner (see SI Appendix Table S2).

**Table 1:** The emotional effect of terrorism: baseline results.

	Negative (1)	Fear (2)	Anger (3)	Sadness (4)	Disgust (5)
Panel A: Simple specification					
Post-attack	0.003*** (0.000)	0.006*** (0.000)	0.003*** (0.000)	0.002*** (0.000)	0.002*** (0.000)
Mean of DV (pre-attack)	0.115	0.057	0.056	0.059	0.039
Observations	7,643,102	7,643,102	7,643,102	7,643,102	7,643,102
Number of users	323,992	323,992	323,992	323,992	323,992
Panel B: Interaction with a 24-hour bandwidth					
Post-attack	0.000 (0.000)	0.002*** (0.000)	0.001*** (0.000)	0.000 (0.000)	0.001*** (0.000)
24-hour bandwidth	– 0.000 (0.000)	– 0.000 (0.000)	0.000 (0.000)	0.000 (0.000)	0.000 (0.000)
Post-attack $\times$ 24-hour bandwidth	0.010*** (0.001)	0.012*** (0.000)	0.008*** (0.000)	0.005*** (0.001)	0.004*** (0.000)
Mean of DV (pre-attack)	0.115	0.057	0.056	0.059	0.039
Observations	7,643,102	7,643,102	7,643,102	7,643,102	7,643,102
Number of users	323,992	323,992	323,992	323,992	323,992
Individual $\times$ attack FEs	$\checkmark$	$\checkmark$	$\checkmark$	$\checkmark$	$\checkmark$
Hour FEs	$\checkmark$	$\checkmark$	$\checkmark$	$\checkmark$	$\checkmark$
Tweet-level controls	$\checkmark$	$\checkmark$	$\checkmark$	$\checkmark$	$\checkmark$

The dependent variable (DV) is the sentiment or emotion shown in the first row. Time window: 3 days before, the same day, and 3 days after each attack. The tweets are aggregated at the minute level. $\textit{Post-attack}$ is a binary variable that takes value 1 if the tweet was posted after the minute of the attack, and 0 otherwise. $\textit{24-hour bandwidth}$ is a binary variable capturing the 24 hours before and the 24 hours after each attack. Standard errors are clustered at the individual-level and reported in parentheses. * $p<.10$; ** $p<.05$; *** $p<.01$

The overall public mood in social media adjusts very quickly to new events and information^44^. This raises the possibility that the emotional reactions following a terrorist attack may be influenced by subsequent government activities, communications, or other unobserved factors, rather than the attack itself. Failing to isolate the impact of terrorism threat itself leads to the problem of “compound treatment”^45^, which can undermine the quality and consistency of inferences. To address this issue, we estimate the post-attack effects on a narrower sample of tweets posted 24 hours before and after each attack. As shown in panel B of Table 1, all negative emotions are substantially more intense in the few hours after an attack (as captured by the interaction of the treatment variable with a 24-hour bandwidth), and then return to baseline levels in the following days (as captured by the treatment variable alone). Furthermore, fear and anger appear again to be the dominant negative emotions, with estimates suggesting that tweets posted 24 hours after an attack contain 21% more fear and 14% more anger compared to those posted 24 hours prior to the attack.

**Fig. 1:**
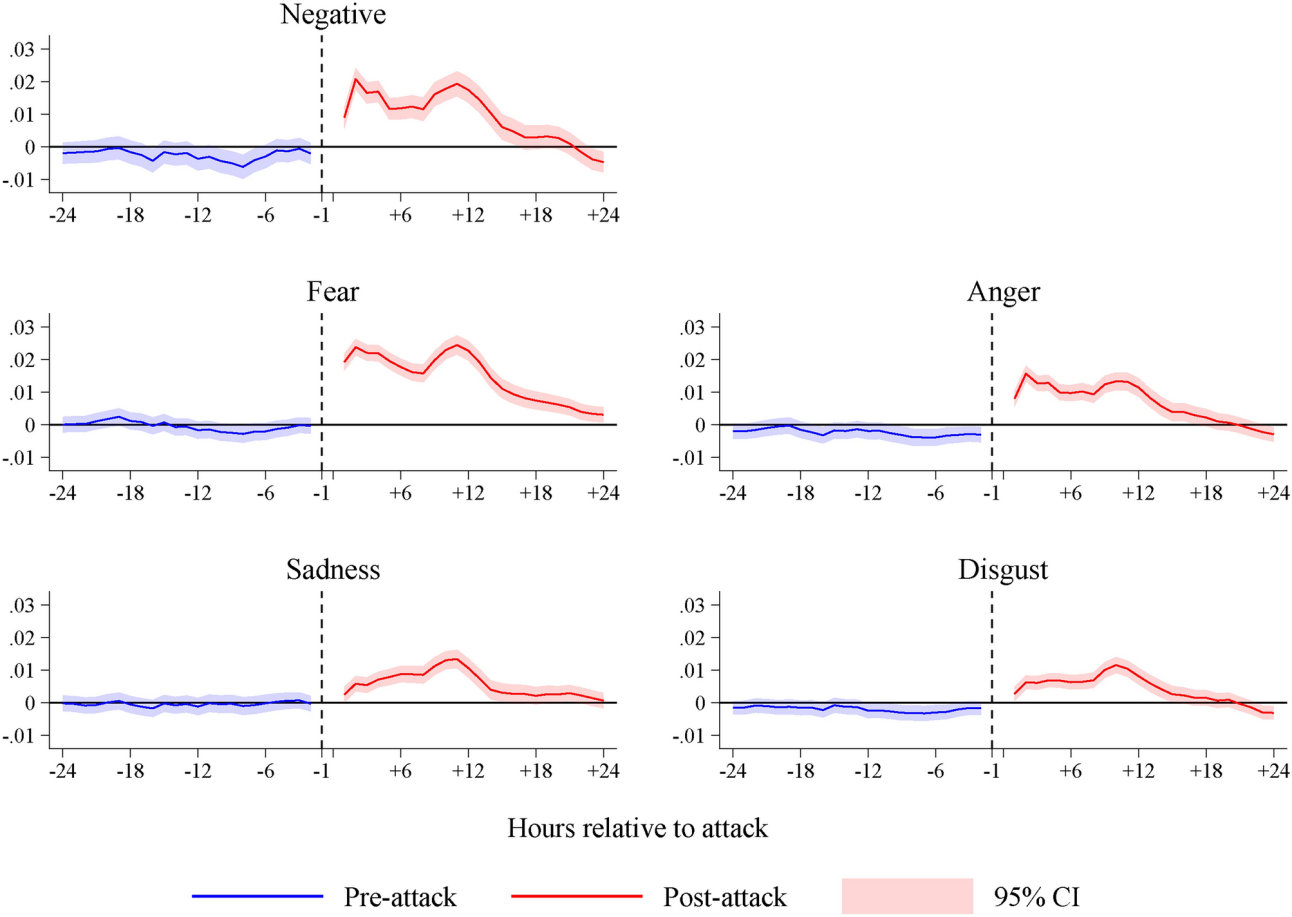
The emotional effect of terrorism: time-to-event analysis. *Notes:* The figure shows the evolution of negative feelings 24 hours before and 24 hours after the sampled attacks. The tweets are aggregated at the hour level. The blue (red) solid line shows the 3-hour moving average estimates before (after) the attacks, taking the hour before the attack as the baseline. The tweets posted in the hour after the attack are dropped from the estimations. The shaded areas show the 95 percent confidence intervals.

To provide further insights on the temporal dynamics of the emotional effects, we aggregate the tweets at the hourly level and replace the treatment variable with time indicators representing each hour before and after the selected attacks. Figure 1 illustrates the 3-hour moving average estimates of these indicators, with the hour before the attack serving as the baseline. The post-attack emotional reactions can be classified into two groups: fear and anger, which exhibit an immediate surge and gradually decrease over time; and sadness and disgust, which slightly increase and persist at that level until about 14 hours after the attack. It is worth noting that the emotional content of tweets posted 1-24 hours before the attacks does not appear to differ significantly from that of tweets posted 1 hour before the attacks, indicating the absence of pre-existing trends. This is also corroborated when we examine an extended version of this figure based on the full time window (see SI Appendix Figure S.2).

To what degree do the observed effects stem from tweets that mention terrorism? To answer this question, we run the same regressions after restricting the sample to include non-terror-related tweets; that is, we exclude tweets that contain the word ‘terror’ and/or other related terms, as identified using a Word2Vec algorithm. Table 2 presents the results for this sub-sample, based on the specification with the 24-hour bandwidth. The estimates are very similar to those reported in Table 1, indicating that people experience negative emotional reactions in all areas of online discourse following a terrorist attack. In other words, exposure to terrorist violence affects individuals’ overall emotional state and the language they use to express their thoughts, even when they are not explicitly discussing terrorism-related issues or the incident itself.

**Table 2:** The emotional effect of terrorism: non-terror-related tweets.

	Negative (1)	Fear (2)	Anger (3)	Sadness (4)	Disgust (5)
Post-attack	– 0.001 (0.000)	0.001*** (0.000)	0.000*** (0.000)	– 0.000 (0.000)	0.000*** (0.000)
Post-attack $\times$ 24-hour bandwidth	0.008*** (0.001)	0.010*** (0.000)	0.006*** (0.000)	0.003*** (0.001)	0.003*** (0.000)
Non-terror-related obs.	7,584,596	7,584,596	7,584,596	7,584,596	7,584,596
Individual $\times$ attack FEs	$\checkmark$	$\checkmark$	$\checkmark$	$\checkmark$	$\checkmark$
Hour FEs	$\checkmark$	$\checkmark$	$\checkmark$	$\checkmark$	$\checkmark$
Tweet-level controls	$\checkmark$	$\checkmark$	$\checkmark$	$\checkmark$	$\checkmark$

See notes for Table 1. The variable $\textit{24-hour bandwidth}$ is included in all estimations. Non-terror-related tweets are those that do not include the word ‘terror’ and/or any related terms, as identified using a Word2Vec algorithm; i.e., extremism; jihad; islamist; islamic; radical; militants; suicide/bomb; bombing; terrror; teror; isis; isil; far-right. Standard errors are clustered at the individual-level and reported in parentheses. * $p<.10$; ** $p<.05$; *** $p<.01$

### Heterogeneity analysis

The magnitude of emotional responses is not expected to be uniform across all eight events. In this section, we examine heterogeneity in the effects with respect to two attack characteristics that have been linked to heightened threat perceptions and increased negative emotions: the motivation of the attacker (i.e., whether the attack is motivated by Islamic extremism) and the number of victims. Additionally, we explore differences in emotional responses based on the amount of media coverage an attack receives, which can be used as a proxy for the event’s relevance and national significance. This is because the media tend to give more attention to attacks that are perceived as more consequential and threatening to the general public^46^.

**Fig. 2:**
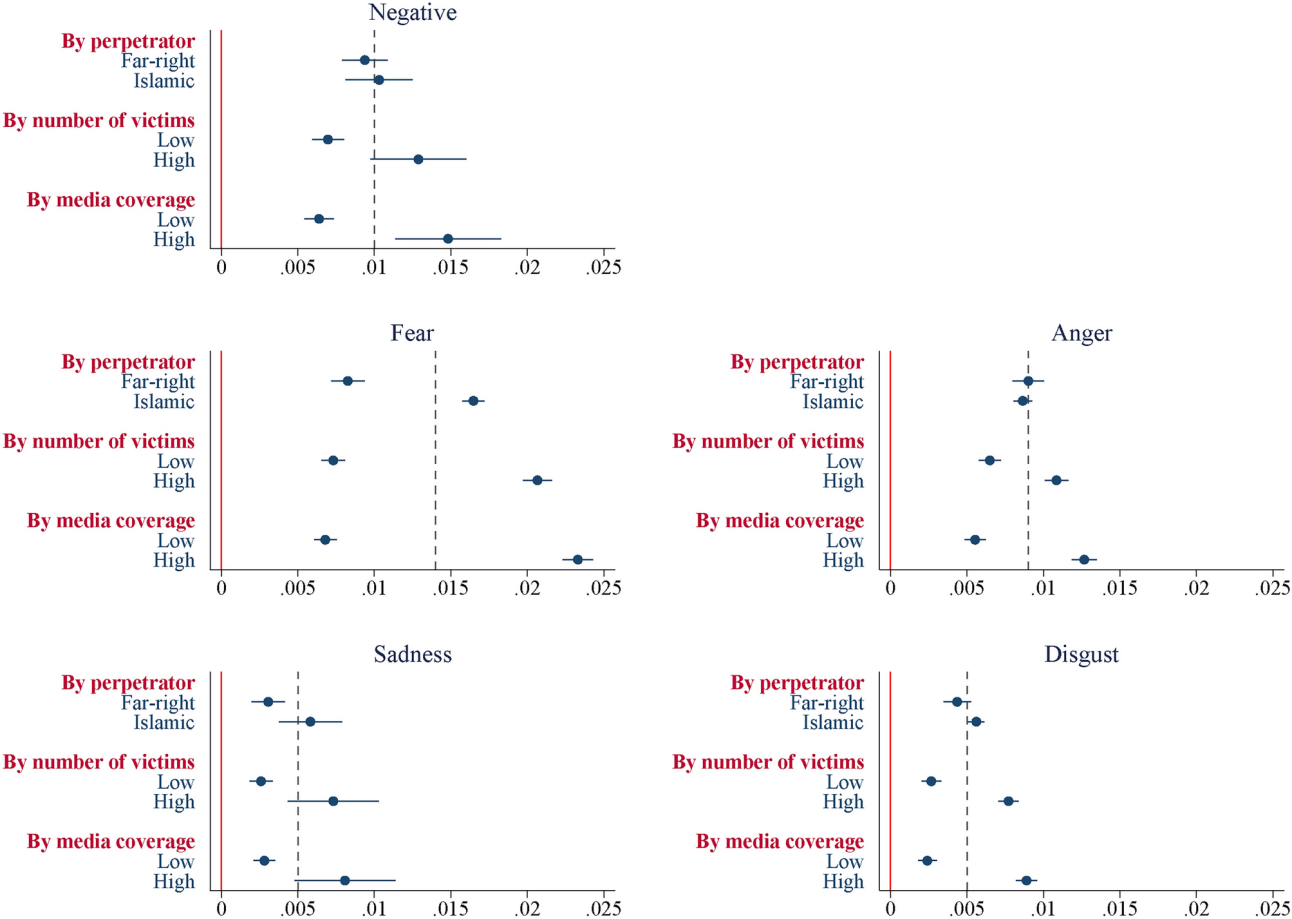
The emotional effect of terrorism: heterogeneity analysis. *Notes:* The figure shows the post-attack estimates for the 24-hour bandwidth (based on the specification in panel B of Table 1) when we run separate regressions for the attack groups displayed on the vertical axis. The horizontal lines signify the 95% confidence intervals of the corresponding estimates.

Figure 2 presents the post-attack estimates for the 24-hour bandwidth (based on the specification in panel B of Table 1) when we run separate regressions for the following attack groups: (i) the six attacks with Islamist perpetrators versus the two attacks with far-right perpetrators; (ii) the four attacks with the highest number of victims (as indicated by the number of deaths and injuries) versus the remaining four attacks; and (iii) the four attacks with the highest media coverage (as measured by the number of LexisNexis hits in the week following the attack) versus the remaining four attacks. Two key conclusions emerge from this analysis. First, attacks motivated by a radical interpretation of Islam result in more fearful sentiments than far-right attacks. This can be attributed to the fact that the former attacks are generally perceived as posing a more systematic threat to national security and democratic values. Second, attacks with a high number of victims and extensive media attention elicit significantly more negative sentiment and emotional responses than those with relatively fewer victims and less media coverage. The difference in effects is substantial across all outcome variables, but it is particularly pronounced for the fear content in tweets, which is three to four times higher for high-victim / high-coverage attacks.

Due to high correlation between the three conditioning factors (see SI Appendix Table S3), one has to be very cautious in prioritising and uncovering links among them. Nevertheless, the analysis here clearly indicates that the emotional effect of terrorism is stronger for attacks that are deemed more threatening or consequential than others.

### Second-order effects

So far, we have focused on the ‘first-order effects’ of terrorism; that is, the emotions that are triggered by the attack itself. In this section, we examine one of the ‘second-order echo effects’ of terrorism, its impact on emotions towards immigration. Extant research has shown how, after terrorist attacks, members of the broader audience tend to distance themselves from strangers and out-groups in general, and to develop negative attitudes and feelings towards immigrants^22,47^.

**Fig. 3:**
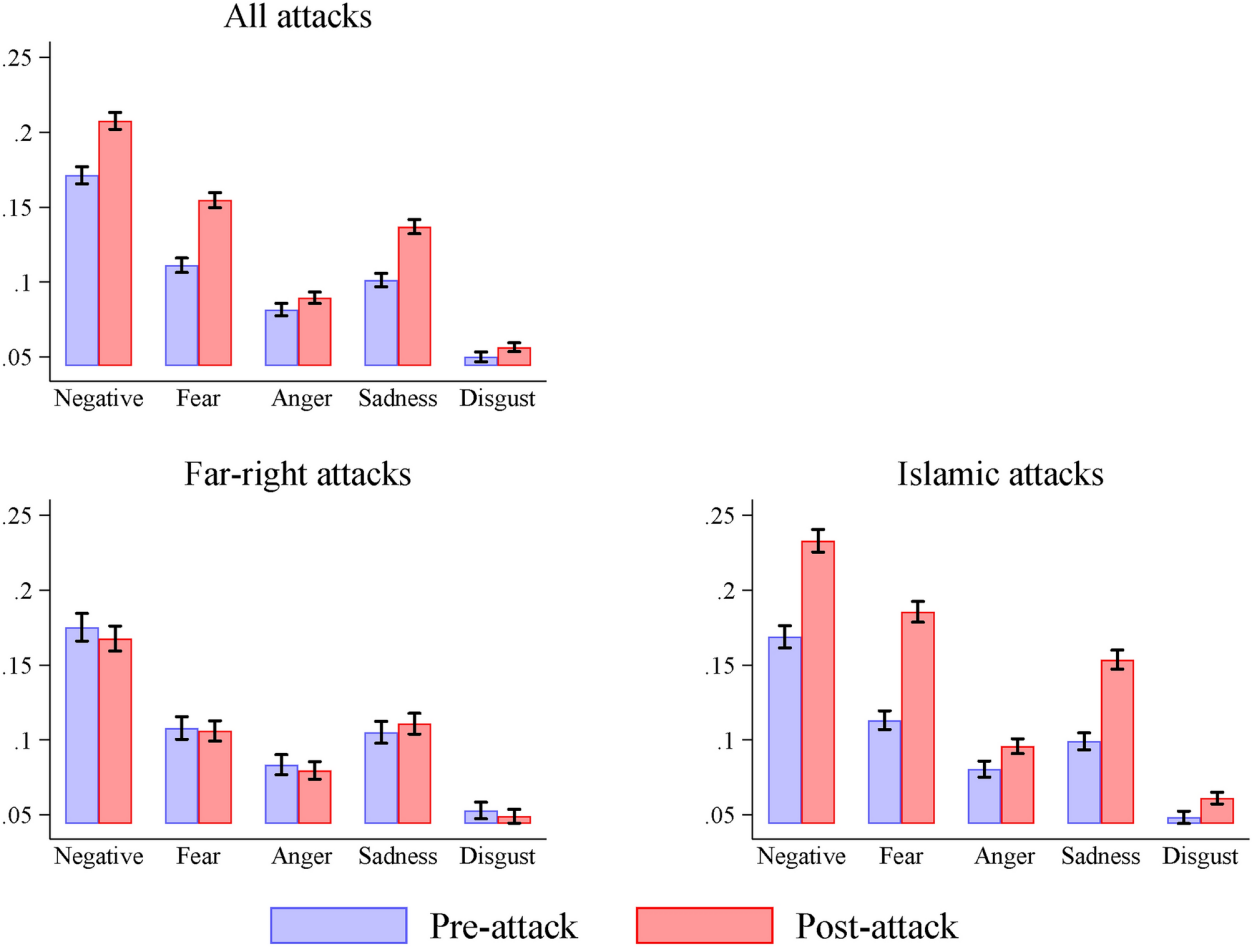
The emotional content of immigration-related tweets. *Notes*: This figure shows the pre- and post-attack average values of the negative sentiment and emotions about immigration. The analysis is based on tweets that include the word ‘immigration’ and other related terms, as identified using a Word2Vec algorithm; i.e., migrant, deport, illegals, undocumented, refugee, citizenship, visa, illegal alien, expedited removal, asylum seeker, as well as typos of the word ‘immigration’. Black bars denote the standard error of the mean.

To accomplish this, we analyse the emotional content of tweets related to immigration; i.e., tweets containing the word ‘immigration’ and other related terms identified by a Word2Vec algorithm. We consider the period of three days before and after each attack (including the day of the attack). Figure 3 compares the pre- and post-attack average values of the negative sentiment and emotions about immigration, calculated using the share of words assigned to a given sentiment/emotion across all lexicon-identified words included in the immigration-related tweets. As shown in the upper panel of the figure, there is a notable increase in negative feelings about immigration following terrorist attacks, particularly in the overall valence, fear, and sadness.

These differences are expected to be influenced by the identity of the perpetrator. Previous research has shown that, following Islamic attacks, the general public is more likely to view foreigners and out-groups as a threat to the homogeneity of the nation-state population^48–50^. Conversely, after far-right attacks, people may soften their views towards immigrants to avert any association with the ideology of the perpetrator, and to show support for the perceived victims of violence^51^. To test for this, we compare the pre- and post-attack emotional content of immigration-related tweets separately for Islamic attacks and far-right attacks. The results confirm the above expectations: while there is a substantial increase in negative sentiment and emotions about immigration following the six Islamic attacks (by 50% to 100%), the corresponding effects for the two far-right attacks are in the opposite direction, albeit small in magnitude (see the lower panels of Figure 3).

In Table 3, we address the same question using regression analysis. In panels A and B, we present the results for all attacks and Islamic attacks, respectively, by including attack fixed effects, in addition to hour fixed effects and tweet-level controls (i.e., number of retweets, replies, likes and quotes). This approach enables us to compare emotional reactions about immigration around the same attack, while controlling for unobserved factors related to time and other tweet characteristics. Panels C and D present the results when we replace attack fixed effects with individual $\times$ attack fixed effects, which allows us to exploit within-individual variation around the same attack. The latter specification is useful as it enables more robust causal inferences, but it has the disadvantage of reducing the statistical power of our analysis, as only a small number of individuals post immigration-related tweets before and after the same attack. Regardless of the specification used, we observe a large and highly statistically significant increase in negative feelings about immigration after Islamic attacks, with fear (about immigration) being the emotion that displays the largest and most persistent post-attack rise, in line with the patterns observed in Figure 3.

**Table 3:** Terrorism and the emotional content of immigration-related tweets.

	Negative (1)	Fear (2)	Anger (3)	Sadness (4)	Disgust (5)
Panel A: All attacks; includes attack FEs					
Post-attack	0.019*** (0.005)	0.027*** (0.005)	0.000 (0.004)	0.023*** (0.004)	0.001 (0.003)
Post-attack $\times$ 24 hour bandwidth	0.069*** (0.010)	0.059*** (0.009)	0.029*** (0.007)	0.047*** (0.008)	0.019*** (0.005)
Pre-attack dependent variable	0.172	0.112	0.082	0.102	0.050
Observations	15,979	15,979	15,979	15,979	15,979
Number of users	8,453	8,453	8,453	8,453	8,453
Panel B: Islamic attacks, includes attack FEs					
Post-attack	0.040*** (0.007)	0.051*** (0.007)	0.005 (0.005)	0.037*** (0.006)	0.006 (0.004)
Post-attack $\times$ 24 hour bandwidth	0.089*** (0.012)	0.070*** (0.011)	0.038*** (0.008)	0.057*** (0.010)	0.022*** (0.006)
Pre-attack dependent variable	0.169	0.113	0.081	0.099	0.048
Observations	9,849	9,849	9,849	9,849	9,849
Number of users	5,781	5,781	5,781	5,781	5,781
Panel C: All attacks; includes individual $\times$ attack FEs					
Post-attack	0.017 (0.011)	0.012 (0.009)	– 0.007 (0.007)	0.009 (0.009)	– 0.008 (0.006)
Post-attack $\times$ 24 hour bandwidth	0.034* (0.018)	0.038** (0.015)	0.028** (0.012)	0.016 (0.015)	0.029*** (0.009)
Pre-attack dependent variable	0.177	0.113	0.084	0.102	0.049
Observations	8,085	8,085	8,085	8,085	8,085
Number of users	1,859	1,859	1,859	1,859	1,859
Panel D: Islamic attacks; includes individual $\times$ attack FEs					
Post-attack	0.031** (0.014)	0.036*** (0.013)	– 0.003 (0.010)	0.011 (0.012)	0.000 (0.007)
Post-attack $\times$ 24 hour bandwidth	0.045** (0.023)	0.037* (0.020)	0.030* (0.016)	0.028 (0.019)	0.024* (0.012)
Pre-attack dependent variable	0.179	0.117	0.082	0.104	0.044
Observations	4,545	4,545	4,545	4,545	4,545
Number of users	1,162	1,162	1,162	1,162	1,162
Hour FEs	$\checkmark$	$\checkmark$	$\checkmark$	$\checkmark$	$\checkmark$
Tweet-level controls	$\checkmark$	$\checkmark$	$\checkmark$	$\checkmark$	$\checkmark$

See notes for Table 1. The analysis is based on tweets that include the word ‘immigration’ and other related terms, as identified using a Word2Vec algorithm; i.e., migrant, deport, illegals, undocumented, refugee, citizenship, visa, illegal alien, expedited removal, asylum seeker, as well as typos of the word ‘immigration’. Standard errors are clustered at the individual-level and reported in parentheses. * $p<.10$; ** $p<.05$; *** $p<.01$

### Further analyses and robustness tests

We probe the robustness of the main results in a number of auxiliary analyses, which are all reported in detail in SI Appendix.

In Section B.1, we test the sensitivity of our results to using restricted samples of Twitter users: those who are present in our dataset before and after all sampled attacks; and those who posted the same number of tweets before and after a given attack. Overall, our inferences do not change: once again, we find that the tweets posted 24 hours after the attacks convey more negative feelings than those posted 24 hours before the attacks. This guards against the concern of selection bias due to terrorist attacks being correlated with Twitter users’ engagement with the platform or the frequency of their posts.

In Section B.2, we perform a number of tests to strengthen our causal inference and rule out possibility of a spurious relationship. First, we focus on one of the most important attacks in our sample (the 2017 Westminster attack) and set the attack date to be 1 week prior to the actual date. Second, we benchmark our results against a failed and not immediately reported attack: the 2017 assassination attempt of PM Theresa May. Third, we examine the treatment effect on outcomes that should not be affected by terrorist events; namely, people’s feelings about the weather. In all cases, we find no evidence of significant spikes in negative emotions in the aftermath of the incidents. As a further test, we perform Monte Carlo permutation tests that randomly shuffle the data 500 times and estimate a treatment effect for each random draw. The permuted data produce estimates which are lower than those in Table 1, suggesting that there is 0% probability that the observed effects are observed by chance.

In Sections B.3 and B.4, we examine the treatment effect on positive emotions – using the Emolex sentiment analysis tool – and check robustness to using alternative tools (VADER, Textblob, and composite indices). Overall, the patterns observed are in line with our previous findings and do not seem to be influenced by the method we use to measure sentiment.

In Section B.5, we explore the conditionality of effects upon geographic proximity, as captured by the proximity in kilometers between the user’s geo-tagged location and the attack location. The analysis suggests that, while physical proximity can play a moderating role in how individuals respond to terrorism, this role is rather weak. This is likely due to the severity and emblematic nature of the attacks in our sample.

Finally, in Section B.6, we estimate our model separately for each of the eight individual attacks. In all cases, we find evidence of heightened negative emotions in the aftermath of the incidents, suggesting that our results are not driven by a small subset of the sampled attacks. In line with our previous results, we also find that the effects are stronger and statistically more robust for attacks with a high number of victims, widespread media coverage, and Islamist perpetrators. This is also verified in Section B.7, where we consider the temporal dynamics of the emotional effects (using time-to-event analysis) for the different groups of attacks.

## Discussion

Terrorist attacks trigger strong emotional responses that affect well-being, attitudes and behaviour. In this study, we investigate the emotional impact of eight major terrorist attacks that occurred in the UK between 2016 and 2020 on the sentiments and emotions expressed in tweets. We provide evidence of dramatic spikes in negative emotions in the immediate wake of the incidents, particularly in fear and anger. As such, terrorists do seem to achieve the purpose of inciting fear, intimidation and spreading psychological distress far beyond the immediate victims^52,53^. These effects persist when we focus on tweets that are unrelated to terrorism, indicating the far-reaching and pervasive effects of terrorist acts on public sentiment.

Our results challenge the notion that European audiences have become desensitised to terrorism due to its frequency in recent years^54,55^. Rather, we find that the discrete emotional responses are conditioned by the characteristics of the attacks. For instance, Islamic attacks elicit a stronger fear response from the public than far-right attacks. This may be partly due to the media and policymakers framing Islamic attacks as the work of organised terrorist cells, while portraying right-wing attacks as isolated, ‘lone wolf’ incidents^56,57^.

Our findings also reveal a significant post-attack increase in negative feelings about immigration. Notably, these effects are exclusively driven by Islamic attacks, supporting the notion that terrorism fuels discussions about Muslim immigration^4^. This can result in increased anti-immigrant sentiment, possibly due to heightened suspicion towards perceived out-groups^22,47,58^. Public anxieties and security concerns following terrorist acts can significantly influence ongoing immigration debates, potentially leading to stricter immigration policies^47,59^ and increased polarisation within society^60^. Negative social media content about immigration can also lead to offline actions, such as anti-refugee incidents^42^ and violence against minority groups^43^.

There are two notable limitations in our study, leaving key areas for further investigation. First, our focus on eight prominent attacks within a single country provides a snapshot, but not the whole picture. Examining a wider range of incidents, and including more frequent, less sensational attacks, would offer a more comprehensive understanding of terrorism’s emotional toll. Second, the geographical scope of the study is limited. As information travels instantaneously through social media, the emotional repercussions of a major attack in one country crosses national borders, potentially impacting citizens in geographically distant nations. Future studies could explore how terrorism in one region can influence the emotional well-being of individuals in other, seemingly unaffected parts of the world.

A growing body of scientific literature interrogates the effects of societal challenges – such as climate change and Covid-19 – on emotional responses and well-being^61,62^. The availability of vast amounts of Twitter data has allowed researchers to monitor large-scale emotional changes in real-time, offering valuable insights for public health campaigns^63^. Part of this body of research’s aim is to provide policymakers the tools to develop evidence-based strategies and interventions. To actively respond to terrorist threats, policymakers could work with health professionals to devise a package of well-being support that can be deployed in the aftermath of an attack. There is also scope for officials in communication roles to “get ahead of the curve” by signalling well-being support and delivering reassuring messaging from the first few minutes after an attack.

Taken together, our findings add emphasis to the argument that terrorist incidents should be treated as instances of public ‘trauma’ which shape collective perceptions of insecurity and vulnerability^31^. As such, understanding the emotional effect of terrorism is important for policymakers tasked with managing the public’s ‘collective trauma’ and building resilience in the aftermath^64^.

## Methods

### Data and variables

Twitter is the second highest ranking social media website in the UK (behind Facebook), and in April 2020, its monthly social network market share in the country was around 37 percent. Contrary to other social networks, a large percentage of the messages posted by Twitter users (tweets) are freely accessible. Moreover, the platform provides geographic information about the tweets (geotags), which can be used to analyse emotions and attitudes at some sub-national level^65^.

Users of Twitter can comment news and communicate their views on current events from their own accounts in *real time*^33^. As such, contrary to questionnaires, analysing Twitter data allows us to track changes in an individual’s attitudes and emotions within very short time intervals – e.g., every few minutes or hours – which can help to produce valid causal estimates of theoretically relevant shocks. In addition, it allows us to study a person’s emotions and attitudes based on their own language and frames of reference, rather than their responses to survey questions, which are inherently subject to some misinterpretation and bias. For instance, as stressed by the literature on response, survey questions do not only measure public opinion; they can also shape and channel it by the manner in which they frame issues, order the various alternatives, and set the context^66^.

We use Twitter’s API V2 to obtain English language tweets with a geotag in the UK. We sample tweets that were posted around the timing of eight major terrorist incidents: the murder of MP Jo Cox in June 2016, the Westminster attack in March 2017, the Manchester Arena bombing in May 2017, the London Bridge attack in June 2017, the Finsbury Park attack in June 2017, the Parsons Green bombing in September 2017, the London Bridge stabbings in November 2019, and the Reading stabbings in June 2020. These are considered to be the most salient (domestic) attacks that occurred over the period 2016-2020: all eight attacks resulted in fatalities or a large number of injuries and received widespread national media coverage. This implies that, regardless of where each attack occurred, individuals from all over the UK were potentially exposed to them. SI Appendix Section A.1 offers background material on these attacks.

To minimise the possibility of other events driving the estimated effects, we employ a short-range time window around the attacks: 3 days before, the same day, and 3 days after each attack. Also, since we are interested in variation within individuals, we only keep Twitter users with both pre- and post-attack tweets for at least one of the sampled terrorist incidents. This procedure results in a large individual-level unbalanced panel consisting of around 7.6 million observations (24 observations, on average, per individual).

In order to measure the valence and emotional content of the text contained in each tweet, we use a dictionary-based method, the NRC Emotion Lexicon (EmoLex)^67,68^, developed by crowd-sourced manual annotations. While most of the other lexicon-based sentiment analysis tools offer a simple categorisation of sentiment into positive and negative (and occasionally neutral), EmoLex enables further categorisation into a number of specific emotions^69^. This is important for developing an understanding of the distinct emotional micro-foundations driving public opinion about terrorism. EmoLex is prone to the general criticism of lexicon-based approaches, specifically that they are unable to detect different context and multiple meanings of words. However, lexicon-based approaches have the advantage of being more transparent than alternative approaches – such as machine learning tools – and not requiring training on a specific domain^70^.

The EmoLex lexicon contains 14,182 words and 25,000 senses, and each one of these words/senses is linked to two sentiments (negative and positive) and eight emotions (anger, fear, sadness, disgust, anticipation, trust, surprise, and joy). The sentiments are assigned either a value 1 (associated) or a value 0 (not associated); whereas the emotions are assigned a value from 0 to 1, capturing the share of lexicon-identified words/senses in a tweet that are linked to a given emotion. We focus on the negative sentiment and emotions given their strong influence on judgement and choices^14,71^, and their high correlation with offline behaviour – see, e.g., evidence on the relationship between negative tweets about Islam and offline hate crimes^43^. Furthermore, analysing positive emotions in the aftermath of terrorist attacks can be troublesome since the text may also capture words of empathy towards the victims.

Besides the textual content and geotag data, we also retain some additional information about the tweets (number of retweets, replies, likes and quotes), which we introduce in our model to control for heterogeneity with respect to tweet-specific characteristics. More details about the Twitter data collection and coding are presented in SI Appendix Section A.2. Descriptive statistics of all variables used in our analysis are provided in SI Appendix Table S1.

### Identification strategy

Our model specification allows us to exploit variation within individuals, net of potential temporal unobserved factors. More formally, it can be written as follows:

1 $$\begin{aligned} Y_{ijs} = \beta \textit{Post-attack}_{ijs} + \delta \textbf{X}_{ijs} + \vartheta _{ih} + \lambda _{js} + \varepsilon _{ijs} \end{aligned}$$

where $Y_{ijs}$ is the sentiment or emotion linked to tweet *i* posted by individual *j* around attack *s*; $\textit{Post-attack}_{ijs}$ is a binary indicator that takes value 1 if the tweet was posted after the minute of the attack, and 0 otherwise; $\textbf{X}_{ijs}$ is a vector of tweet-level controls, as described above; $\vartheta _{ih}$ represents hour fixed effects (capturing the hour of the day, *h*, that the tweet was posted); $\lambda _{js}$ represents individual $\times$ attack fixed effects; and $\varepsilon _{ijs}$ is an error term, clustered at the individual level. Our parameter of interest, $\beta$, measures the effect of terrorism on the outcome variable, with a positive (negative) value indicating that exposure to terrorism strengthens (weakens) the corresponding sentiment or emotion.

It must be stressed that the inclusion of individual $\times$ attack fixed effects eliminates any time-invariant sources of heterogeneity across individuals, and controls for the possibility that individuals posting tweets before the attacks are systematically different from those posting tweets after the attacks. Thus, rather than comparing the tweets of different individuals and around different attacks, we estimate whether the tweets posted by a given individual after a specific attack convey more negative feelings than those posted by the same individual before the same attack. Furthermore, adding hour fixed effects in Eq. (1) accounts for residual heterogeneity arising from the hour of the day that the tweet was posted (e.g., night hours vs day hours).

As noted above, by employing a short-range time window around the attacks, we can reduce the potential for bias due to other events. Given how quickly the overall public mood in social media changes and adjusts to new information^44^, we also present results when we focus on a narrower time window. To do that, we augment Eq. (1) with a binary variable (*24hr-bandwidth*) capturing the 24 hours before and the 24 hours after each attack, together with its interaction with the *Post-attack* dummy. In this way, we can estimate how individual-specific feelings change in the few hours after the attacks compared to the few hours before the attacks.

Our identification strategy relies on the assumption that the timing of the event in question is exogenous and unexpected. This is clearly the case of violent events, such as the assassination of political leaders or terrorist attacks^45^. A remaining threat to our identification arises from the possibility of selection into tweeting around the attacks. An important reason why this threat is less acute in our context is that we exploit variation within individuals who have at least one tweet both before and after an attack. However, to further ensure that selection in not affecting our results – e.g., when Twitter users systematically change the topic and the frequency of their tweets in the wake of a terrorist incident – we adopt two complementary approaches. First, we run the same regressions for tweets that do not contain terror-related terms (see Results Section). These are identified using the Word2Vec algorithm^72^, which is trained on a Google News dataset containing about 100 billion words. Second, we test whether our results persist when we restrict the sample to include the Twitter users who are present in our dataset before and after all sampled attacks, and those who posted the same number of tweets before and after a given attack (see SI Appendix Section B.1).

## Supplementary Information

Supplementary Information.

## Data Availability

The anonymised data that support the findings of this study and the code used in the analysis will be openly available in Harvard Daraverse upon publication.
