# Supplementary material for: The emotional effect of terrorism
Source: Sci Rep. 2024 Nov 3;14:26525. doi: 10.1038/s41598-024-77350-5 (PMC11532472; doi:10.1038/s41598-024-77350-5)
Supplement: Supplementary file 1 — Supplementary Information. [file 41598_2024_77350_MOESM1_ESM.pdf]

The emotional effect of terrorism

Supplementary Information (SI) Appendix

For Online Publication

Contents

A Additional Information **2**

A.1 Background material on the sampled attacks 2

A.2 Description of Twitter data 9

A.3 Additional tables and figures 11

B Robustness Tests and Further Insights **16**

B.1 Using restricted samples of Twitter users 16

B.2 Further identification validity tests 18

B.3 The impact of terrorism on positive feelings 23

B.4 Alternative approaches of measuring sentiment 24

B.5 The moderating role of geographic proximity 27

B.6 Results for individual attacks 29

B.7 Heterogeneity analysis: time-to-event figures 31

C References **35**

A Additional Information

A.1 Background material on the sampled attacks

We sample tweets that were posted around the timing of eight major terrorist incidents in the UK over the period 2016-2020: (i) the murder of MP Jo Cox in June 2016; (ii) the Westminster attack in March 2017; (iii) the Manchester Arena bombing in May 2017; (iv) the London Bridge attack in June 2017; (v) the Finsbury Park attack in June 2017; (vi) the Parsons Green bombing in September 2017; (vii) the London Bridge stabbings in November 2019; and (viii) the Reading stabbings in June 2020. Below we provide background material on these attacks.

(i) Murder of Jo Cox (June 2016)

Labour party MP Jo Cox was murdered in her constituency of Batley and Spen in Yorkshire, on June 16, 2016, a week prior to the Brexit referendum. The perpetrator, Thomas Mair, was a 53-years-old white supremacist, whose hatred extended to white people he deemed 'collaborators'. Mair's links to far-right movements and his obsession with Nazism, white supremacy and apartheid-era South Africa were well documented,[1] but the attack was not immediately identified as a terrorist incident. The media subsequently labelled Mair a 'far-right terrorist'. Though Mair was trialled for murder, the prosecutors argued that his crimes were "nothing less than acts of terrorism", while the judge noted in delivering Mair's life sentence that his "inspiration was not love of country but admiration for Nazism".[2]

The British public were deeply shocked: Union Flags on public buildings, including the Palace of Westminster, were flown at half mast, and the Brexit referendum campaign was suspended. Prominent UK leaders issued tributes and condolences, including the Conservative Prime Minister, David Cameron, the Labour party leader, Jeremy Corbyn, and MEP Nigel Farage, leader of the UK Independence Party (UKIP) and prominent Leave.EU campaigner.[3] Farage came under scrutiny as during the course of the attack, Mair shouted "Britain First", the name of a far-right organisation aligned with Farag's party and policies. Some commentators and scholars drew connections between the aggressive rhetoric oft-adopted by the Leave campaign, and Mair's motives (Jones, 2020; Bove et al., 2024;

[1]Sources: voxpoliticalonline.com, splcenter.org, and theguardian.com.
[2]Source: bbc.co.uk.
[3]Source: theguardian.com.

Pickard et al., 2023). Farage rebutted these accusations, dismissing the murder as caused by 'one deranged, dangerous individual'.[4] Leaders of far-right groups – including Paul Golding, the leader of Britain First – rushed, at least initially, to distance themselves from the attack,[5] while extreme-right activists' reactions ranged from attributing the attack to Mair's mental-health issues to insinuating the 'truthfulness' of the attack.[6]

(ii) Westminster Attack (March 2017)

On 22 March 2017, 52-year-old Khalid Masood drove a car into pedestrians along Westminster Bridge in London. Masood abandoned the vehicle outside the Palace of Westminster and stabbed a police officer before being shot and killed by police forces.[7] The attack resulted in 5 deaths and at least 50 injuries. Masood was an Islamic extremist, claiming in a Whatsapp message uncovered by security forces that he was waging Jihad in response to Western military action in the Middle East.[8] On 23rd of March the Islamic State of Iraq and the Levant (ISIL), announced that the attacker was "a soldier of the Islamic State, executing the operation in response to calls to target citizens of coalition nations".[9] The Metropolitan Police, immediately designating the attack a terrorist incident, investigated these claims but found that Masood had acted alone, though he was inspired by ISIL rhetoric. Masood had previously been investigated as a peripheral figure in an MI5 investigation of a 2010 terror plot, but a risk assessment in that instance found that he posed no threat.[10]

The attack met with condemnation across the political spectrum. The prime-minister, Theresa May, attributed the attack to 'Islamic ideology', characterising it as an attempt to 'silence our democracy' while emphasising that Masood was not an active target of investigation by intelligence services despite his prior involvement in a terror plot.[11] Jeremy Corbyn, leader of the opposition, remarked in the Commons that the attack was an 'appalling atrocity'.[12]

[4]Source: mirror.co.uk.
[5]Source: huffingtonpost.co.uk.
[6]Source: globalcomment.com.
[7]Source: start.umd.edu/gtd.
[8]Source: telegraph.co.uk.
[9]Source: independent.co.uk.
[10]Source: telegraph.co.uk.
[11]Source: bbc.com.
[12]Ibid.

(iii) Manchester Arena Bombing (May 2017)

On 22 May 2017, Salman Ramadan Abedi, a 22-year-old Mancunian man of Libyan descent, detonated a home-made bomb in the foyer of Manchester Arena, as people were leaving an Ariana Grande concert. Twenty-three attendees – six of them children – died in the explosion,[13] and 1,017 were injured, 112 of whom required hospitalisation.[14] The attack proved the deadliest episode of terrorism in Britain since the London bombings of July 7, 2005.[15] The government immediately raised the terror threat level to 'critical', the highest level in a five-point scale, before reverting to the pre-existing level ('severe') five days later.[16]

The government pointed to the bombing as part of its motivation for updating its counter-terrorism strategy and introducing new policies such as broader data-sharing between counter-terrorism police and other agencies in 2018.[17] In 2022, testimonies in the Manchester Arena Inquiry reported that MI5 had sufficient intelligence to open an investigation against Abedi as a threat to national security a month prior to the attack. It had failed to do so because the agency was "struggling to cope" with increasing workload and could not carefully consider of the case.[18] British newspapers reported widely on the attack with graphic, emotional coverage. The Daily Mail shared 'horrifying videos' from inside the arena, as "terrified concert-goers flee for their lives",[19] while several newspapers emphasised the presence of children among the victims. PM Theresa May condemned how the attacker saw a "room packed with young children as an opportunity for carnage".[20] Data from LexisNexis confirms the relevance of the bombing in public debate: it is indeed the most widely covered attack in the 2016-2020 period, with over 11,900 results during the first month after the event.[21]

[13]Source: bbc.co.uk.
[14]Source: files.manchesterarenainquiry.org.uk.
[15]Source: kerslakearenareview.co.uk.
[16]Source: bbc.co.uk.
[17]Source: assets.publishing.service.gov.uk.
[18]Source: theguardian.com.
[19]Source: dailymail.co.uk.
[20]Source: gov.uk.
[21]Keywords ('terrorist' OR 'terrorism') AND 'Manchester' AND 'arena' were used to identify news reports about the attack.

(iv) London Bridge Attack (June 2017)

On the 3rd of June, 2017, a van was driven at pedestrians on London Bridge, then crashed on Borough High Street.[22] Three perpetrators, wearing fake suicide vests, exited the vehicle and began stabbing civilians around a cluster of restaurants and pubs along Stoney Street.[23] The assailants were shot dead by armed officers of the Metropolitan police Special Firearms Command. The attack resulted in 8 deaths, and 48 injuries.[24] In the days following the attack, numerous news stories emerged which vividly detailed how members of the public and a number of unarmed police officers had attempted to intervene.[25] On the 4th of June, ISIL claimed responsibility for the attack,[26] but no link between the assailants and the group could be confirmed by authorities.[27] The perpetrators were identified as Khuram Shazad Butt, a Pakistan-born British citizen, Rachid Redouane, a failed asylum seeker residing in Dagenham, and Youssef Zaghba, a Moroccan and Italian dual-national residing in East London.[28] Two of the assailants had been identified by various authorities as connected to Islamic extremism before the attacks.[29] Butt was a known member of the banned extremist group Al-Muhajiroun and was investigated in relation to his involvement with suspects involved in the July 2005 London bombing,[30] while Zaghba had been previously identified by the Italian authorities as a terror threat.[31]

National election campaigning was suspended by all political parties in the day following attack, with the controversial exception of UKIP whose leader, Paul Nuttall, claimed that suspending their campaign was "what the extremists would want".[32] Theresa May attributed the attack to 'evil Ideology of Islamic Extremism'.[33] The Mayor of London, Sadiq Khan praised London's 'defiant unity in the face of adversity' while also condemning the spike in hate crimes targeting Muslims in the wake of the attack.[34]

[22]Source: start.umd.edu/gtd.
[23]Ibid.
[24]Ibid.
[25]Source: news.sky.com.
[26]Source: gov.uk.
[27]Source: start.umd.edu/gtd.
[28]Source: theguardian.com.
[29]Source: nytimes.com.
[30]Ibid.
[31]Source: telegraph.co.uk.
[32]Source: theguardian.com.
[33]Source: gov.uk.
[34]Source: theguardian.com.

(v) Finsbury Park Attack (June 2017)

The second attack by a far-right perpetrator in our sample took place on June 19, 2017. A 48-year-old man, Darren Osborne, drove a van into a crowd of Muslims near the Finsbury Park Mosque, in north London, causing one death and injuring ten. Osborne was motivated by his anger over the Islamic attacks in London and Manchester, and a child grooming scandal in Rochdale involving men of Asian origin. The incident was immediately considered a terrorist attack by politicians, counter-terrorism police, and the media.

Most British newspapers' front pages on the following day focused on the perpetrator, rather than the victims, though there were some notable exceptions (e.g., the Guardian and the Independent). Importantly, Osborne's affiliation to far-right groups appeared secondary in reporting, in contrast with the pattern generally observed following Islamic attacks. Nonetheless, media coverage of the incident was rather high, with 1,152 results on LexisNexis within the first month.[35] The attack was generally condemned across the political spectrum: PM Theresa May praised London's multiculturalism and promised a stronger effort against Islamophobia, as did religious leaders from different creeds, while Prince Charles visited the Finsbury Park Mosque to meet community leaders.[36] Yet, some comments on the social media pages of far-right groups such as Britain First suggested that the attack was justified and painted the perpetrator as a 'hero'.[37]

(vi) Parsons Green Bombing (September 2017)

On September 15th, 2017, an explosion occurred on the District line train at Parsons Green Underground station in London, injuring 69.[38] The blast was caused by a homemade 'bucket bomb' packed with the explosive chemical triacetone triperoxide (TATP), which partially exploded.[39] The following day ISIL claimed responsibility for the attack, though the Metropolitan Police cast doubt on this claim.[40] An investigation led by the Metropolitan Police's Counter Terrorism Command immediately ensued,[41] culminating in the arrest of several individuals linked to Islamic extremism by the authorities.[42] Ahmed Hassan was eventually identified as the sole perpetrator, and was later sentenced to life imprison-

[35] Keywords used: ('terrorist' OR 'terrorism') AND 'Finsbury Park'.
[36] Sources: theguardian.com, independent.co.uk, and bbc.co.uk.
[37] Source: thetimes.co.uk.
[38] Source: start.umd.edu/gtd.
[39] Ibid.
[40] Source: bbc.co.uk.
[41] Ibid.
[42] Source: bbc.com.

ment on 23rd March 2018.[43] Hassan had entered the UK illegally via Calais, but successfully sought asylum, despite admitting in his asylum interview that he had spent three months in an ISIS training camp.[44] During the sentencing, it was concluded that Hassan was driven by "a mind-set of ISIS extremism, a deep-seated hatred of this country, a desire for revenge against Britain and America whom he blamed for his father's death in Iraq and anger at the continued bombing of Iraq by Western Coalition forces".[45]

Following the attack, PM Theresa May announced that the terror threat level would be raised to the highest level, 'critical'.[46] By the 17th of September, it was lowered to 'severe', the level previously designated during the months after the Manchester Arena attack.[47] The attack was widely condemned by actors across the political spectrum. Theresa May branded the incident a 'cowardly attack' while the Mayor of London, Sadiq Khan emphasised that London "will never be intimidated or defeated by terrorism".[48]

(vii) London Bridge Stabbings (November 2019)

On 29 November 2019, Usman Khan, a former British prisoner of Pakistani descent convicted of terror offences, stabbed five people inside and outside Fishmongers' Hall, adjacent to London Bridge. Two of the victims died from their stab wounds.[49] Khan, released on license just one year prior on the day of the attack,[50] was attending a conference on offender rehabilitation.[51] After initially threatening to detonate what turned out to be a fake suicide vest, he began stabbing people in the building.[52] Khan then ran outside and stabbed pedestrians on London Bridge, where a civilian eventually managed to restrain him, until the police arrived and shot him dead.[53] ISIL claimed responsibility for the attack, without evidence.[54] In 2021, Independent Reviewer of Terrorism Legislation Jonathan Hall QC, considering Khan's early release, recommended that those who participate in the planning or preparation of terrorist attacks are given automatic life sentences.[55]

[43] Source: judiciary.uk.
[44] Ibid.
[45] Ibid.
[46] Source: bbc.co.uk.
[47] Source: london.gov.uk.
[48] Ibid.
[49] Source: thetimes.co.uk.
[50] Ibid.
[51] Source: bbc.co.uk.
[52] Ibid.
[53] Source: bbc.co.uk.
[54] Source: washingtonpost.com.
[55] Source: richmondandtwickenhamtimes.co.uk.

While investigators concluded that police had lawfully killed Khan,[56] a separate inquiry found that the attacker had not been sufficiently monitored and that the security planning at the event had been sub-par. These factors, the jury concluded, contributed to the death of the two victims.[57]

(viii) Reading Stabbings (June 2020)

On June 20th, 2020, a single assailant with a kitchen knife attacked two groups of civilians at Forbury Park, a public park in the centre of Reading, England.[58] The perpetrator, 25-year-old Khairi Saadallah, was tackled by police called to the scene.[59] The attack resulted in three fatalities, and three serious injuries. Though initial police statements suggested that the motivation for the attacks was unknown,[60] Counter-Terrorism Policing South East, who took over the investigation of the incident in conjunction with MI5, confirmed that the attack was being treated as a 'terror incident'.[61] Investigations confirmed that Saadallah was an Islamic extremist, inspired by ISIL.[62] Saadallah claimed to police that the attack was 'jihad'.[63] It was later uncovered that Saadallah, who had successfully claimed asylum in the UK in after escaping the Libyan Civil War, was a known quantity to MI5, which had obtained evidence that he planned to travel for extremist reasons in 2019.[64] Before the attack, Saadallah was convicted on six occasions for 15 crimes, 8 of which were violent crimes. It was found by Westminster Magistrates court that in Libya, Saadallah had trained and fought for extremist group Ansar Al-Sharia in Libya.[65]

PM Boris Johnson was 'appalled and sickened' by the incident, and hinted at possible legislative action, stating that "if there are lessons that we need to learn about how we handle such case, we will not hesitate to take action where necessary".[66] One of the victim's families subsequently criticised the government for failing to deport Saadallah prior to the attack, despite his violent crime convictions.[67]

---

[56] Source: bbc.co.uk.
[57] Source: standard.co.uk.
[58] Source: start.umd.edu.
[59] Source: independent.co.uk.
[60] Source: thamesvalley.police.uk.
[61] Source: independent.co.uk.
[62] Source: telegraph.co.uk.
[63] Ibid.
[64] Ibid.
[65] Source: news.sky.com.
[66] Source: theguardian.com.
[67] Source: getreading.co.uk.

A.2 Description of Twitter data

We use Twitter API v2 to retrieve data on tweets posted three days before, the same day, and three days after each attack. Focusing on a short-range time window around the attacks allows us to minimise the possibility of other events driving the estimated effects and draw robust causal inferences (Muñoz et al., 2020). In addition, tweet extraction and data processing is very time-consuming – as well as subject to the cap of 10 million tweets per month as part of API v2 – which prevents us from considering a wider time frame, especially in the context of multiple attacks.

We retrieve tweets based on following criteria: (i) they are written in English; and (ii) they include a place or location based in the UK which is tagged by the user. Twitter geographical information comes from two sources: tweet-level geographic metadata and account-level geographic metadata. While account-level geographic data constitutes a substantial 30-40% of all tweets, it is prone to bias as the location is selected by the user; e.g., users can specify incorrect locations (for instance, the information might not be up to date) or fictional locations (for instance, Atlantis). Tweet-specific location information, on the other hand, is retrieved from the tweet (geo-tagged tweets), and can be either specific (based on GPS data), or a Twitter "Place"[68] – which includes the area from which the tweet is posted, or a specific place in this area, which is selected by the user. The most precise location data is determined from geo-tagged tweets, which constitute 1-2% of all tweets. The majority of tweets or profiles do not have any geographical metadata specified, albeit there are various methods and techniques for location prediction (see, e.g., Zheng et al. (2018) for a review). We use geo-tagged tweets that are tagged within the UK, which generates a very large number of tweets (more than 9 million) with precise locations. The corresponding information includes the name of the location, the type of place (country, government administrative unit, city, and point of interest), as well as the bounding box of the tweet. 85.3% of the tweets in our sample have a city as a location, 10.8% have a government administrative unit, 0.7% have a point of interest, 0.2% have a country, and 3.1% do not have a specific location. In addition to the tweet content, the time and location, we also extract data on retweet count, reply count, like count, quote count, and total number of tweets per user. We focus on Twitter users with both pre- and post-attack tweets for at least one of the sampled terrorist incidents, which brings the total number of tweets used in our analysis to 7,643,102.

We use the NRC Emotion Lexicon (EmoLex) (Mohammad and Turney, 2013, 2010) to

[68]These tweets constitute around 80% of all geo-tagged tweets.

measure the sentiment and emotions of tweets. The lexicon contains 14,182 words and 25,000 senses, and each one of these words/senses is linked to two sentiments (negative and positive) and eight emotions (anger, fear, sadness, disgust, anticipation, trust, surprise, and joy). The sentiments are assigned either a value 1 (associated) or a value 0 (not associated); whereas the emotions are assigned a value from 0 to 1, capturing the share of lexicon-identified words/senses in a tweet that are linked to a given emotion. Figure S.1 shows the number and visual proportions of words (in our dataset) associated with each sentiment and emotion.

Figure S.1: Number of words linked to each sentiment and emotion

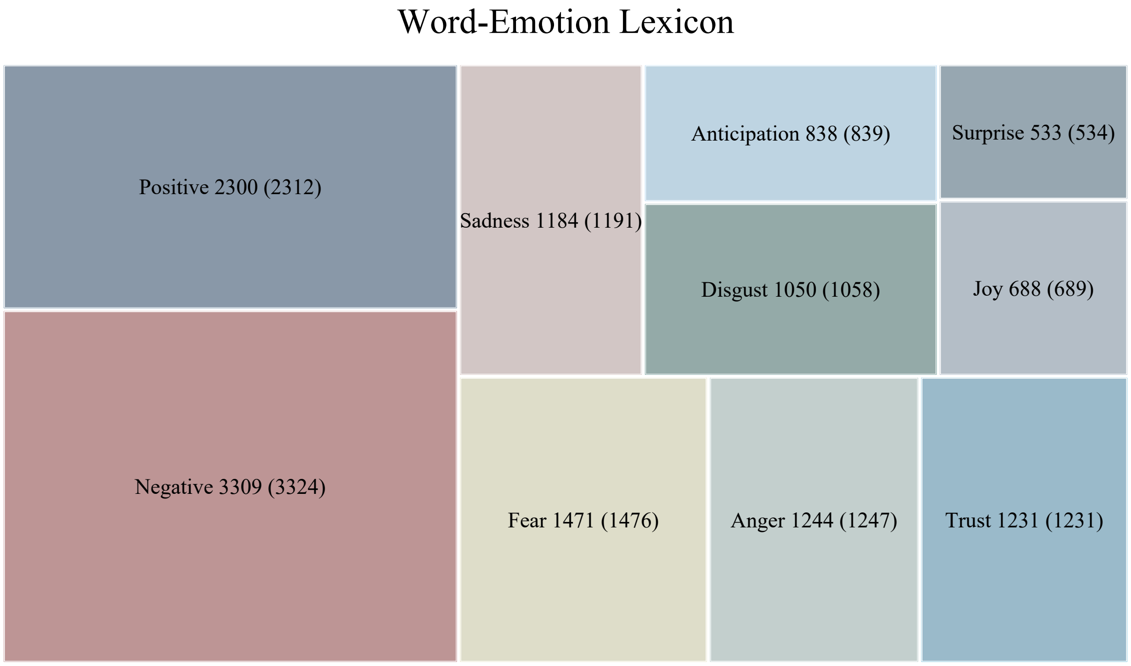

Notes: This figure shows the number and visual proportions of words (in our dataset) associated with each sentiment and emotion. The total number of words in the EmoLex lexicon associated with the corresponding sentiment or emotion is reported in parenthesis.

A.3 Additional tables and figures

- Table S.1 provides descriptive statistics of the variables used in our analysis for the full sample, the treated (post-attack) sample and the control (pre-attack) sample. It also presents the results of t-tests for differences in means across the pre- and post-attack groups.

- Table S.2 examines the sensitivity of the baseline results to alternative model specifications. We adopt an 'incremental strategy', where we start from a simple specification that includes the treatment (post-atack) variable and individual $\times$ attack fixed effects, and we then add hour fixed effects, the tweet-level controls and individual-level error clustering in a progressive manner, until we reach the full specification of panel A in Table 1. As shown in columns (1)-(4), the treatment effects for all outcome variables retain the size and statistical significance throughout these specifications. Finally, in column (5), we can see how the results change once we introduce an interaction term with a 24-hour bandwidth, as in panel B of Table 1.

- Figure S.2 presents an extended version of the time-to-event analysis in Figure 1 based on the full time window (from 3 days before to 3 days after the attacks). This rejects, once again, the presence of pre-existing patterns: the emotional content of tweets posted 1-93 hours before the attacks is very similar to that of tweets posted 1 hour before the attacks (the baseline hour). The figure also confirms that the heightening of negative feelings in the aftermath of the attacks lasts for about 24 hours. This can arguably capture the *direct* effect of terrorism violence on people's emotional state; i.e., before they are exposed to subsequent (related) activities and communication, or other unrelated events.

- Table S.3 shows the attack characteristics and the classification used for the heterogeneity analysis in the *Results* section.

Table S.1: Descriptive statistics and balancing tests

	Full sample mean (sd)	Pre-attack mean (sd)	Post-attack mean (sd)	Difference (3)-(2) p-value
	(1)	(2)	(3)	(4)
Negative	0.12	0.11	0.12	
	(0.24)	(0.24)	(0.24)	0.00
Fear	0.06	0.06	0.06	
	(0.18)	(0.17)	(0.18)	0.00
Anger	0.06	0.06	0.06	
	(0.18)	(0.18)	(0.18)	0.00
Sadness	0.06	0.06	0.06	
	(0.17)	(0.17)	(0.18)	0.00
Disgust	0.04	0.04	0.04	
	(0.15)	(0.15)	(0.15)	0.00
Retweet count	0.74	0.72	0.76	
	(46.29)	(36.49)	(54.33)	0.16
Reply count	0.37	0.37	0.37	
	(3.70)	(2.88)	(4.36)	0.23
Like count	2.79	2.65	2.92	
	(146.31)	(93.95)	(184.25)	0.01
Quote count	0.07	0.06	0.07	
	(4.42)	(4.63)	(4.20)	0.22
Number of tweets	76.52	76.53	76.51	
	(379.21)	(378.21)	(380.21)	0.95
Observations	8,079,246	4,033,469	4,045,777	8,079,246

Notes: This table shows the mean values and standard deviations (in parentheses) of the variables used in our analysis, as well as the results of t-tests for differences in means across the pre- and post-attack groups.

Table S.2: The emotional effect of terrorism: alternative model specifications

	(1)	(2)	(3)	(4)	(5)
Panel A: Negative					
Post-attack	0.003***	0.003***	0.003***	0.003***	0.000
	(0.000)	(0.000)	(0.000)	(0.000)	(0.000)
24-hour bandwidth					–0.000
					(0.000)
Post attack × 24-hour bandwidth					0.010***
					(0.001)
Panel B: Fear					
Post-attack	0.006***	0.006***	0.006***	0.006***	0.002***
	(0.000)	(0.000)	(0.000)	(0.000)	(0.000)
24-hour bandwidth					–0.000
					(0.000)
Post-attack × 24-hour bandwidth					0.012***
					(0.000)
Panel C: Anger					
Post-attack	0.004***	0.003***	0.003***	0.003***	0.001***
	(0.000)	(0.000)	(0.000)	(0.000)	(0.000)
24-hour bandwidth					0.000
					(0.000)
Post-attack × 24-hour bandwidth					0.008***
					(0.000)
Panel D: Sadness					
Post-attack	0.002***	0.002***	0.002***	0.002***	0.000
	(0.000)	(0.000)	(0.000)	(0.000)	(0.000)
24-hour bandwidth					0.000
					(0.000)
Post-attack × 24-hour bandwidth					0.005***
					(0.001)
Panel E: Disgust					
Post-attack	0.002***	0.002***	0.002***	0.002***	0.001***
	(0.000)	(0.000)	(0.000)	(0.000)	(0.000)
24-hour bandwidth					0.000
					(0.000)
Post-attack × 24-hour bandwidth					0.004***
					(0.000)
Individual × attack FEs	✓	✓	✓	✓	✓
Hour FEs		✓	✓	✓	✓
Tweet-level controls			✓	✓	✓
Error clustering at individual level				✓	✓

Notes: Time window: 3 days before, the same day, and 3 days after each attack. The tweets are aggregated at the minute level. *Post-attack* is a binary variable that takes value 1 if the tweet was posted after the minute of attack, and 0 otherwise. *24-hour bandwidth* is a binary variable capturing the 24 hours before and the 24 hours after each attack. Standard errors are clustered at the individual-level and reported in parentheses. * $p < .10$; ** $p < .05$; *** $p < .01$.

Figure S.2: The effect of the attack for 3 days post attack (extended)

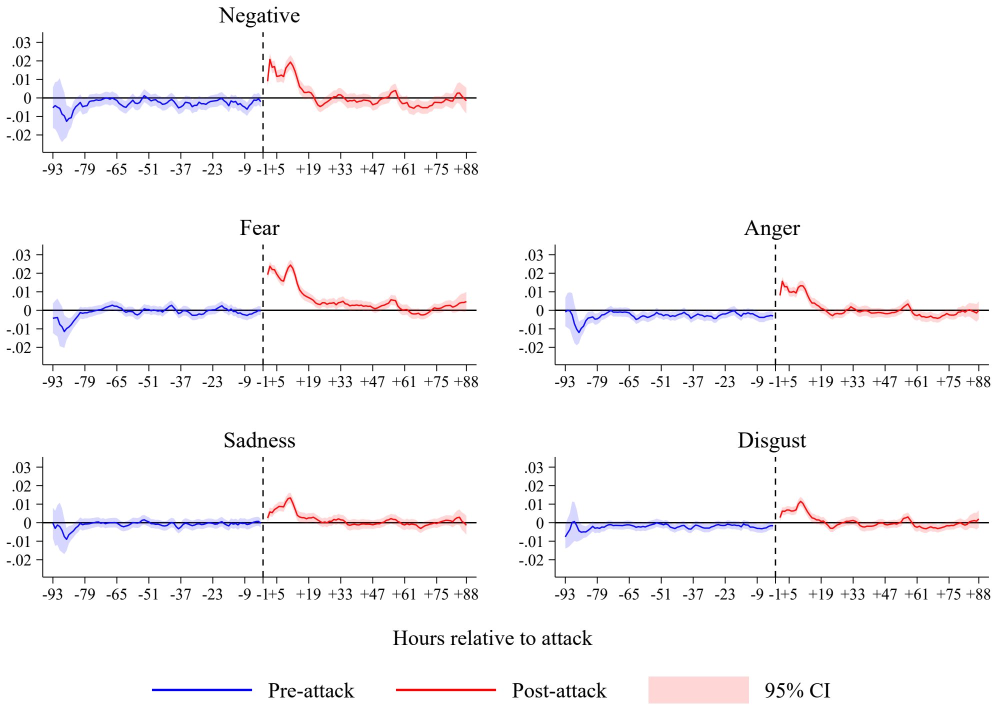

Notes: The figure shows the evolution of negative feelings from 3 days before to 3 days after the sampled attacks. The tweets are aggregated at the hour level. The blue (red) solid line shows the 3-hour moving average estimates before (after) the attacks, taking the hour before the attack as the baseline. The tweets posted in the hour after the attack are dropped from the estimations. The shaded areas show the 95 percent confidence intervals.

Table S.3: Attack characteristics

	Perpetrator type<br>(1)	Number of victims<br>(2)	Number of newspaper articles<br>(3)
Murder of MP Jo Cox in June 2016	Far-right	2	68
Westminster attack in March 2017	Islamic	55 †	4300 †
Manchester Arena bombing in May 2017	Islamic	141 †	11906 †
London Bridge attack in June 2017	Islamic	56 †	4551 †
Finsbury Park attack in June 2017	Far-right	12	3474 †
Parsons Green bombing in September 2017	Islamic	69 †	1282
London Bridge stabbings in November 2019	Islamic	5	2692
Reading stabbings in June 2020	Islamic	6	472

Notes: † indicates that the attack is classified as a high-victim / high-coverage attack.

B Robustness Tests and Further Insights

B.1 Using restricted samples of Twitter users

A possible threat to our identification arises from the possibility of selection into tweeting or changing the topic and the frequency of posts in the aftermath of a terrorist incident. An important reason why this threat is less acute in our context is that we exploit variation within individuals who have at least one tweet both before and after an attack. Furthermore, as shown in *Results* section, our results hold when we run regressions for tweets that do not contain terror-related terms. To further address this concern, we perform two additional checks. First, we restrict the sample to include the users who are present in our dataset before and after all sampled attacks. Second, we only keep the users who posted the same number of tweets before and after a given attack. Table S.4 reports the results when we run the same regression set-up as in panel A of Table 1 using these two 'restricted' samples. Overall, our inferences do not change: once again, we find that the tweets posted 24 hours after the attacks convey more negative feelings than those posted 24 hours before the attacks, and that fear and anger are the emotions that display the largest and more persistent post-attack rise.

Table S.4: The emotional effect of terrorism: using restricted samples of Twitter users

	Negative (1)	Fear (2)	Anger (3)	Sadness (4)	Disgust (5)
Panel A: Users present before and after all attacks					
Post-attack	0.001	0.003***	0.001	0.000	0.001
	(0.001)	(0.001)	(0.001)	(0.001)	(0.001)
Post-attack × 24-hour bandwidth	0.007***	0.011***	0.005***	0.003*	0.002*
	(0.002)	(0.002)	(0.002)	(0.002)	(0.001)
Pre-attack dependent variable	0.112	0.058	0.051	0.061	0.036
Observations	259,423	259,423	259,423	259,423	259,423
Number of users	980	980	980	980	980
Panel B: Users with the same number of tweets before and after a given attack					
Post-attack	0.001	0.001**	0.001	–0.000	0.001*
	(0.001)	(0.001)	(0.001)	(0.001)	(0.000)
Post-attack × 24-hour bandwidth	0.012***	0.014***	0.009***	0.008***	0.006***
	(0.002)	(0.001)	(0.001)	(0.001)	(0.001)
Pre-attack dependent variable (mean)	0.100	0.052	0.047	0.054	0.036
Observations	546,568	546,568	546,568	546,568	546,568
Number of users	119,486	119,486	119,486	119,486	119,486
Individual × attack FEs	✓	✓	✓	✓	✓
Hour FEs	✓	✓	✓	✓	✓
Tweet-level controls	✓	✓	✓	✓	✓

Notes: See notes for Table 1. Standard errors are clustered at the individual-level and reported in parentheses. * $p < .10$; ** $p < .05$; *** $p < .01$.

B.2 Further identification validity tests

In this section, we perform a number of additional tests to strengthen our causal inference.

- First, we consider a placebo treatment during the pre-attack period. More precisely, we focus on one of the most important attacks in our sample (the 2017 Westminster attack) and set the placebo attack date to be 1 week prior to the actual date. Figure S.3 displays the evolution of negative feelings 24 hours before and 24 hours after the placebo attack, based on the same time-to-event analysis as in Figure 1. The patterns show no leaps after the simulated attack, while the occasional peaks before and after the 'event' are short-lived and are characterised by wide confidence intervals.

- Second, we benchmark our baseline results against a failed and not immediately reported attack. To do so, we perform the same analysis as before but we now compare the tweets posted around the 2017 assassination attempt of PM Theresa May, which was confirmed by the media one week later.[69] As can be seen in Figure S.4, the treatment effects are close to zero and statistically insignificant throughout.

- Third, we examine the treatment effect on outcomes that should not be affected by terrorist incidents; namely, people's feelings about the weather. To do that, we create a sample of weather-related tweets (tweets containing the word 'weather') and compare their emotional content around the eight sampled attacks. Figure S.5 presents the evolution of negative feelings about the weather 24 hours before and 24 hours after the attacks. As expected, there is no evidence of significant spikes in the aftermath of the incidents.

- Finally, to ensure that the baseline estimates are unlikely to be observed by chance, we perform Monte Carlo permutation tests that randomly shuffle the data 500 times and estimate a treatment effect for each random draw. The resulting distributions are displayed in Figure S.6. In all cases, the permuted data produce estimates which are lower than those reported in Table 1 (panel A), suggesting that there is 0% probability that the observed treatment effects are observed by chance.

[69]On the 28th November 2017, an Islamist extremist, Naa'imur Zakariyah Rahman, planned to bomb the gates of 10 Downing Street, kill guards and then attack Theresa May with a knife or gun. The suspect was arrested in London after collecting a suicide vest and a fake bomb from undercover operatives.

Figure S.3: Placebo test based on an earlier cut-off point

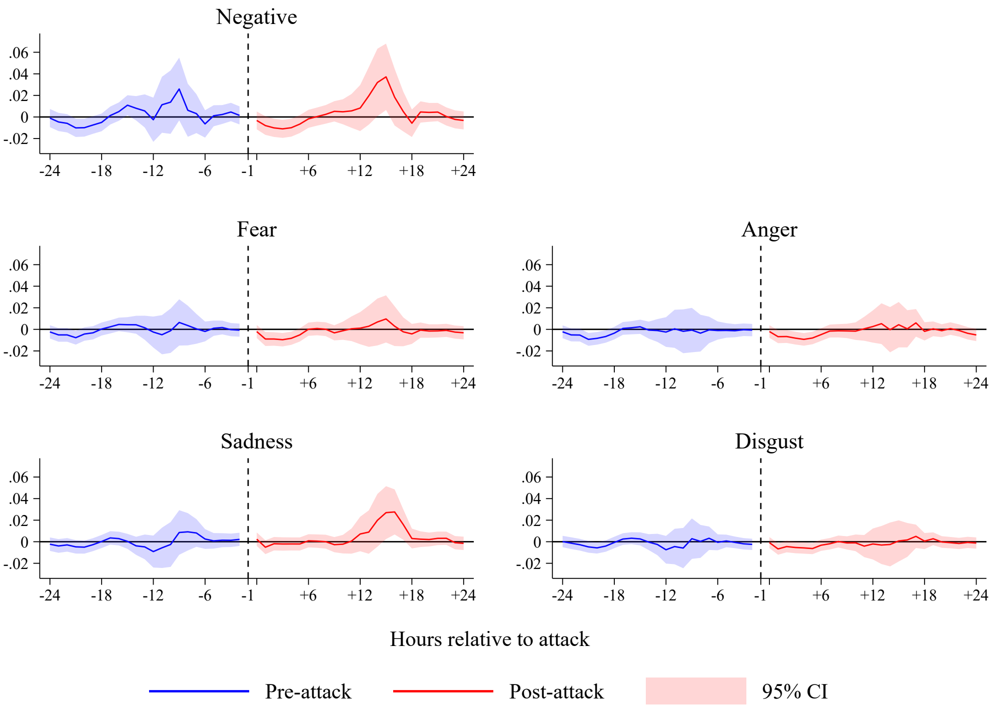

Notes: The figure shows the evolution of negative feelings 24 hours before and 24 hours after the placebo attack. To plot this figure, we rely on a sample of 1,095,903 tweets posted 3 days before and 3 days after the placebo attack date (i.e., 1 week before the 2017 Westminster attack). The tweets are aggregated at the hour level. The blue (red) solid line shows the 3-hour moving average estimates before (after) the attacks. The shaded areas show the 95 percent confidence intervals.

Figure S.4: Comparison with a failed attack

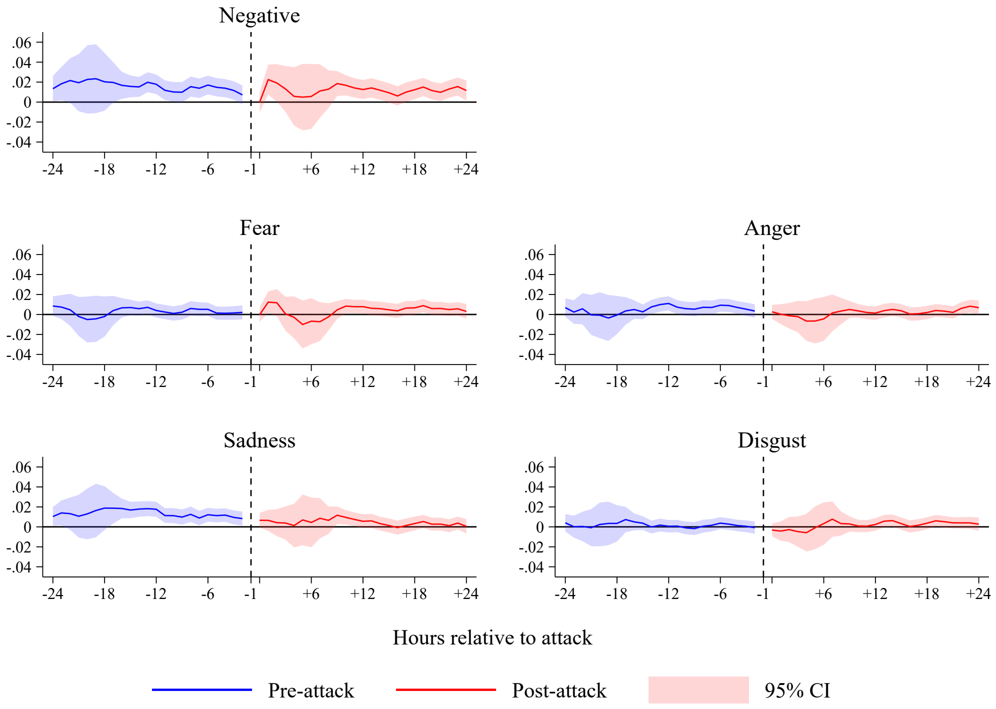

Notes: The figure shows the evolution of negative feelings 24 hours before and 24 hours after the 2017 assassination attempt of PM Theresa May. To plot this figure, we rely on 867,551 tweets posted three days before and three days after the failed attack. We omit the tweets posted on day of the attack, as the time of the attempted assassination and the time of the arrest were not reported. The tweets are aggregated at the hour level. The blue (red) solid line shows the 3-hour moving average estimates before (after) the attacks. The shaded areas show the 95 percent confidence intervals.

Figure S.5: Placebo test based on unrelated outcomes:
feelings about the weather

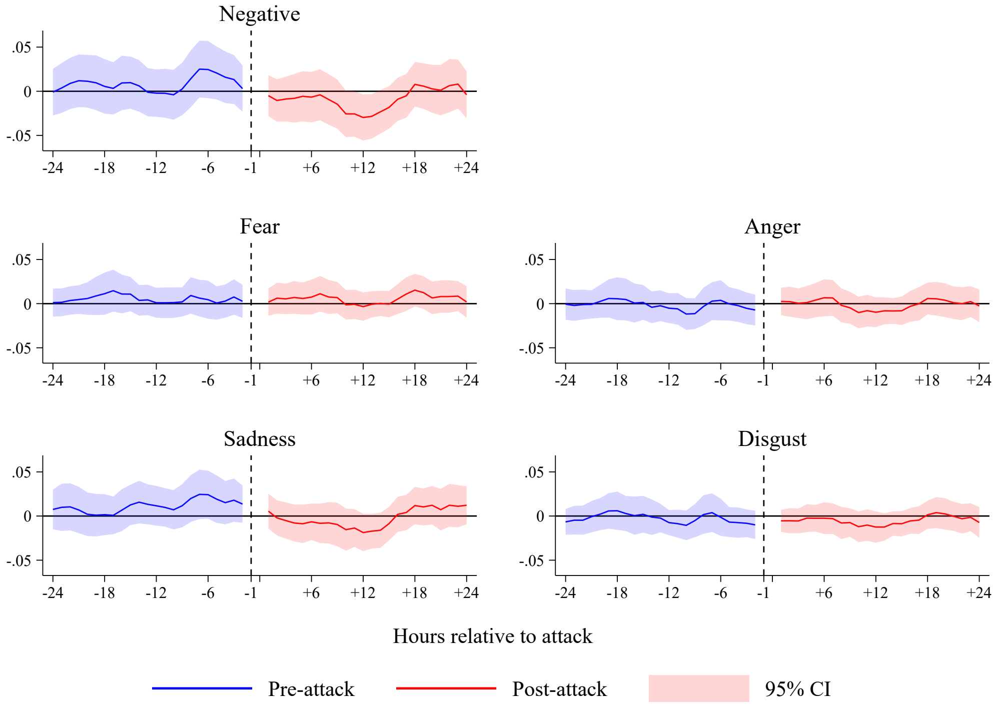

Notes: The figure shows the evolution of negative feelings about the weather 24 hours before and 24 hours after the sampled attacks. To plot this figure, we rely on a sample of 67,940 weather-related tweets posted from 3 days before up to and including 3 days after the attacks. The tweets are aggregated at the hour level. The blue (red) solid line shows the 3-hour moving average estimates before (after) the attacks, taking the hour before the attack as the baseline. The tweets posted in the hour after the attack are dropped from the estimations. The shaded areas show the 95 percent confidence intervals.

Figure S.6: Permutation effect estimates

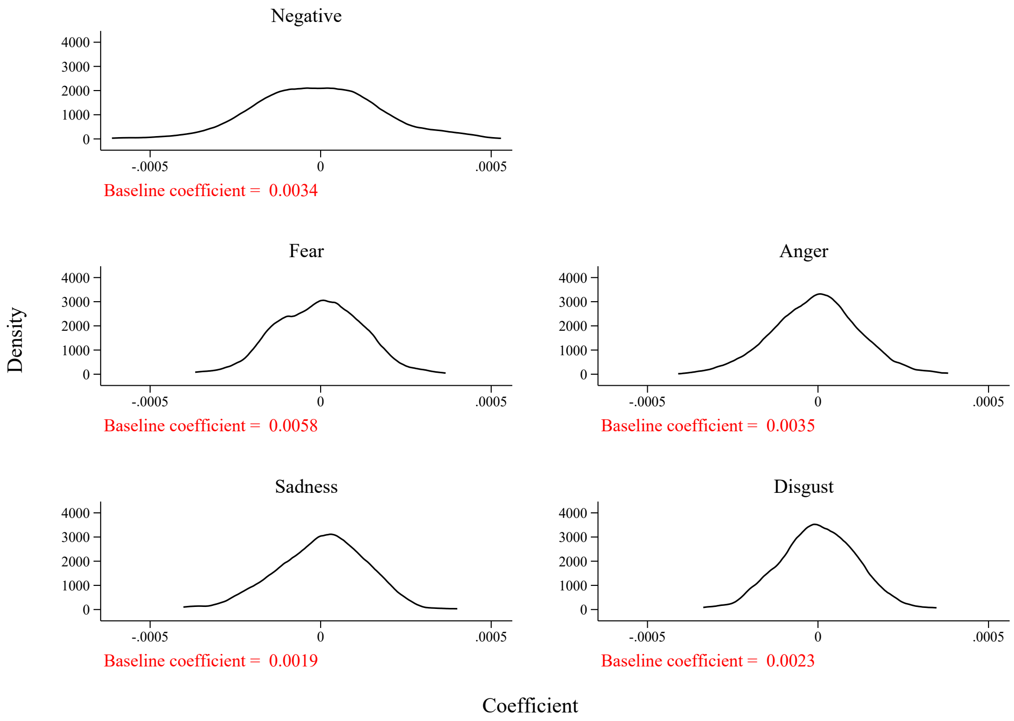

Notes: The figure shows the results of Monte Carlo permutation tests that randomly shuffle the data 500 times and estimate a treatment effect for each random draw. In all cases, the permuted data produce estimates which are lower than those reported in Table 1 (panel A).

B.3 The impact of terrorism on positive feelings

In this section, we examine how positive feelings respond to terrorist attacks. To do so, we carry out the same time-to-event analysis as in Figure 1, but we now focus on the overall positive sentiment. The patterns are displayed in Figure S.7. Generally speaking, we observe the opposite patterns to those of the overall negative sentiment – though the corresponding effects appear to be smaller in magnitude and shorter-lived. This is most likely due to the overall negative effect being counterbalanced by sympathy and compassion towards the attack victims.

Figure S.7: The emotional effect of terrorism: positive sentiment

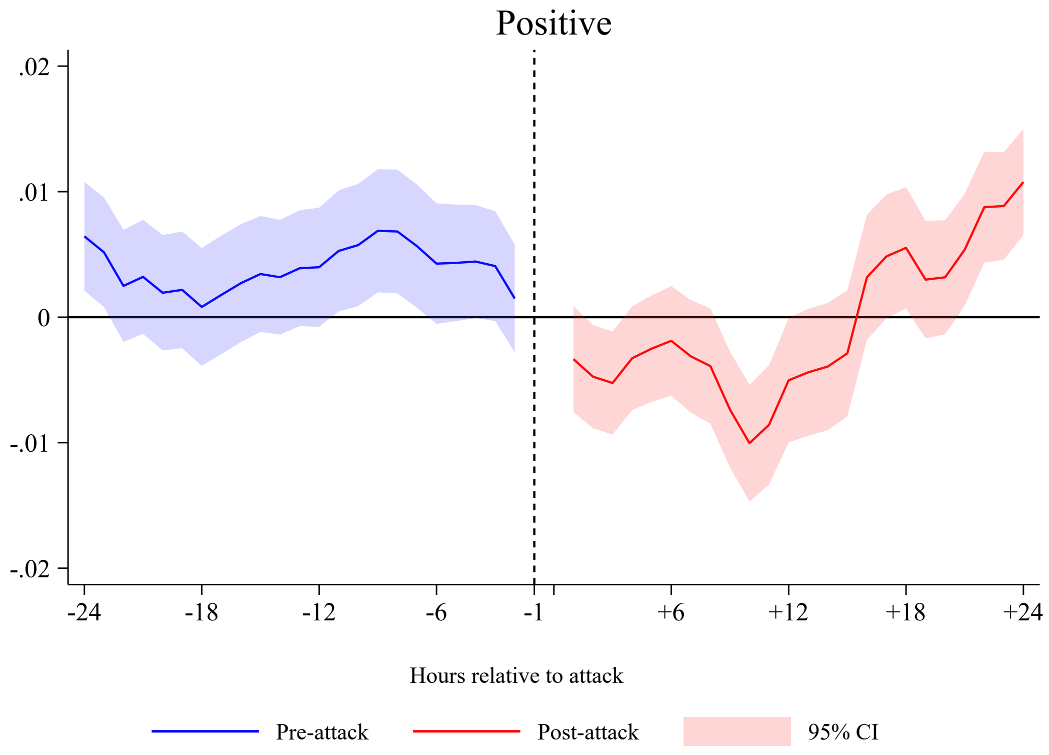

Notes: The figure shows the evolution of positive feelings 24 hours before and 24 hours after the sampled attacks. The tweets are aggregated at the hour level. The blue (red) solid line shows the 3-hour moving average estimates before (after) the attacks, taking the hour before the attack as the baseline. The tweets posted in the hour after the attack are dropped from the estimations. The shaded areas show the 95 percent confidence intervals.

B.4 Alternative approaches of measuring sentiment

So far, we have measured the valence and emotional content of the text contained in tweets using the NRC Emotion Lexicon (EmoLex). In this section, we test the sensitivity of our results to using alternative methods.

First, we replicate our analysis using VADER and Textblob. VADER (Hutto and Gilbert, 2014) is a lexicon- and rule-based sentiment analysis tool, and it is specifically attuned to sentiments expressed in social media. It returns negative, neutral and positive scores as the proportion of text that falls in each category, and a compound score that is computed by summing the corresponding scores of each word in the lexicon, adjusted according to the VADER grammatical and syntactical rules, and then normalised to be in the range between -1 (extreme negative) and +1 (extreme positive). Textblob (Loria et al., 2018) is another lexicon-based sentiment analysis tool, and its analyser returns the sentiment in the form of polarity and subjectivity scores. We consider the polarity score, which falls within the range [-1.0, +1.0], where -1 signifies negative sentiment and +1 positive sentiment. Second, we consider a composite index based on EmoLex. This is calculated by the difference between positive and negative sentiments, weighted by the ratio of the number of words in the tweet that are present in EmoLex to the total number of words in the tweet (LexRatio).

Figure S.8 shows the results of time-to-event analysis for the three VADER scores (positive, negative and neutral); whereas Figure S.9 shows the corresponding results for three alternative composite indices (EmoLex-based index, VADER compound score, and TextBlob polarity score), all capturing a net positive score. Across these figures, there is a sharp drop in neutral, positive, and net positive scores (or a sharp increase in the negative score) just right after the attacks, which persists for about 12 hours and is then followed by a gradual return to baseline levels within the next 12 hours. Overall, the patterns observed are in line with our previous findings and do not seem to be influenced by the method we use to measure sentiment.

Figure S.8: The emotional effect of terrorism: VADER scores

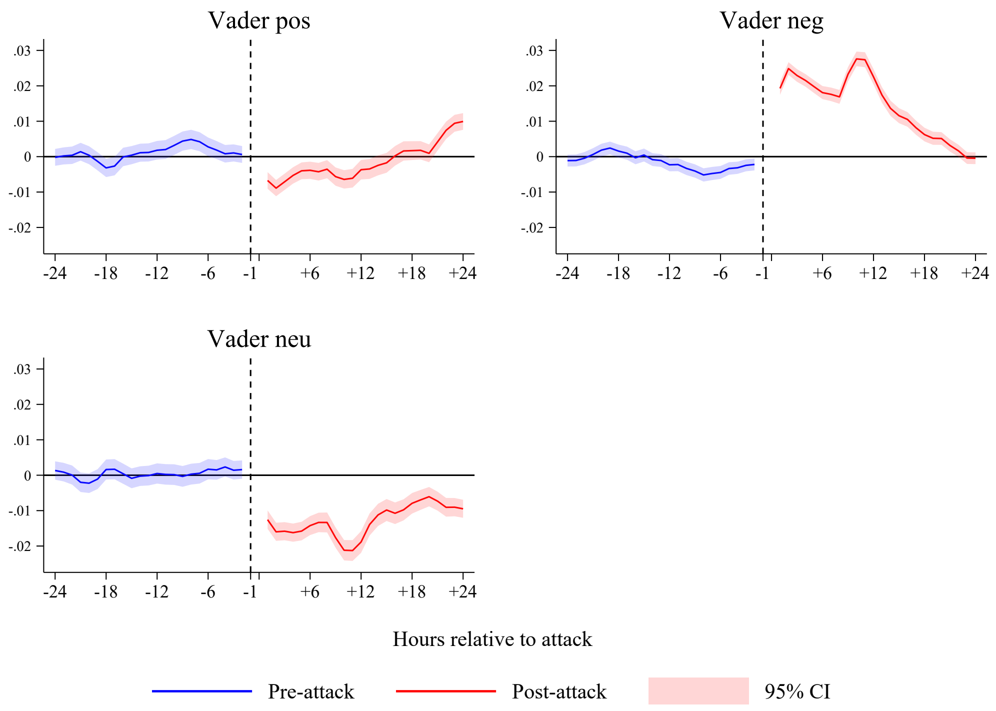

Notes: The figure shows the evolution of the VADER scores (positive, negative and neutral) 24 hours before and 24 hours after the sampled attacks. The tweets are aggregated at the hour level. The blue (red) solid line shows the 3-hour moving average estimates before (after) the attacks, taking the hour before the attack as the baseline. The tweets posted in the hour after the attack are dropped from the estimations. The shaded areas show the 95 percent confidence intervals.

Figure S.9: The emotional effect of terrorism:
alternative composite (net positive) indices

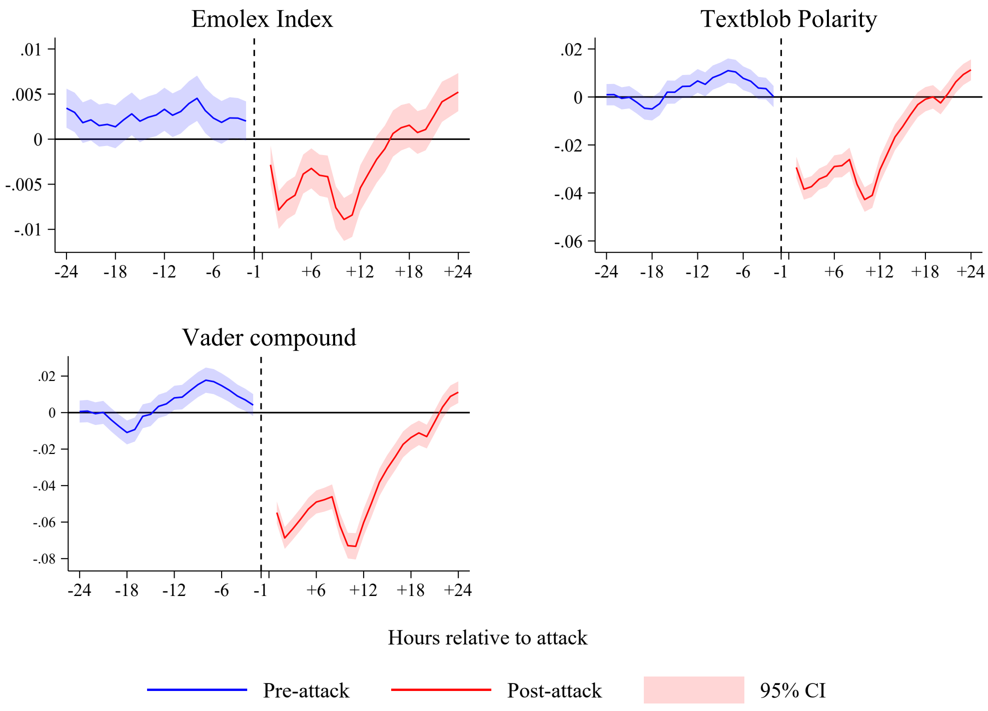

Notes: The figure shows the evolution of three alternative composite indices (all capturing a net positive score) 24 hours before and 24 hours after the sampled attacks. The tweets are aggregated at the hour level. The blue (red) solid line shows the 3-hour moving average estimates before (after) the attacks, taking the hour before the attack as the baseline. The tweets posted in the hour after the attack are dropped from the estimations. The shaded areas show the 95 percent confidence intervals.

B.5 The moderating role of geographic proximity

Geographic proximity to a terrorist incident can amplify the perception of threat and the personal sense of vulnerability, increase mortality salience as individuals feel more connected to the environment where the attack occurred, and affect the extent to which the event is covered by the local media (Nussio et al., 2021; Bove et al., 2022). Following these arguments, one would expect that proximity to terrorism will act as a moderating factor whereby individuals that reside closer an attack are more likely to exhibit negative emotions. To test for this, we interact our treatment variable (*Post-attack*) with the physical distance between the user's geo-tagged location and the attack location. We normalise the distance measure by splitting it into decile groups, where individuals in group 10 are the most proximate to the attack and those in group 1 are the furthest away. Using the estimates from the model with the interaction term and relying on the 24-hour window before and after the attacks, we calculate the margins of the *Post-attack* variable and plot them over the respective decile values of proximity.

Figure S.10 reports the margins for the negative sentiment and the four negative emotions. The results indicate that the closer the individual's geo-tagged location is from the attack, the stronger the effect is on the outcome variables, which verifies the moderating role of geographic proximity in how individuals respond to terrorism. It should be acknowledged, however, that the estimated effect is positive across all values of proximity and only fails to reach statistical significance when we consider the lowest decile groups for sadness – which points to a rather weak dependence on proximity. This is likely due to the severity and emblematic nature of the attacks in our sample – see also Pickard et al. (2023) for a similar finding.

Figure S.10: The moderating role of geographic proximity

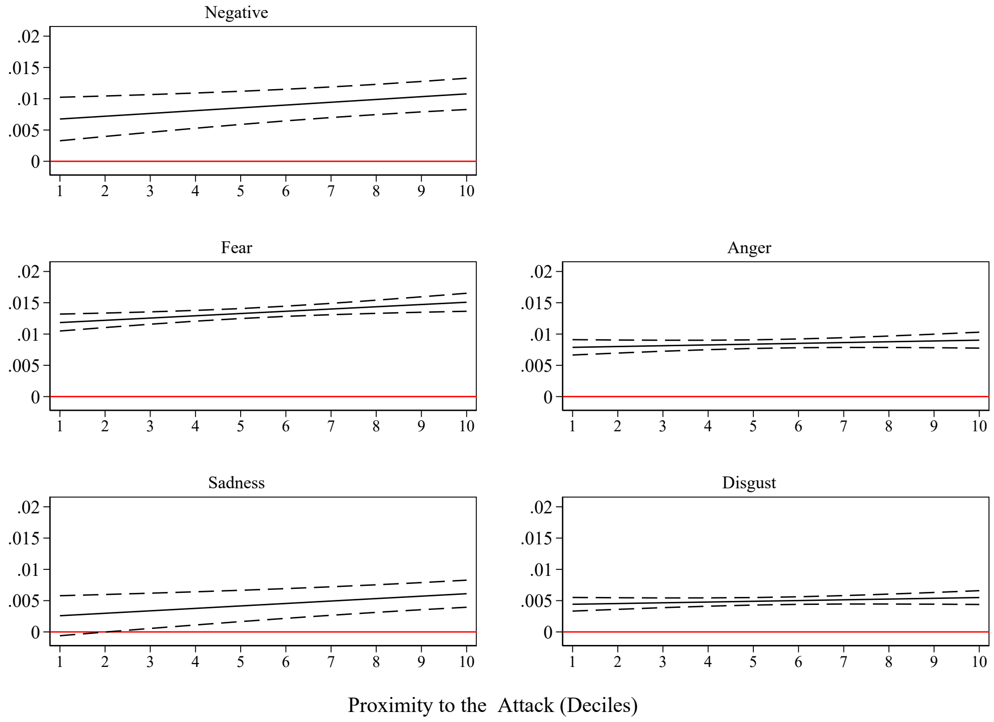

Notes: Proximity to the attack is the kilometer proximity (binned into deciles) between the user's geo-tagged location and the attack location. Dashed lines signify 95% confidence intervals.

B.6 Results for individual attacks

Given the large volume of tweets, we are able to estimate our model separately for each of the eight sampled attacks. Table S.5 presents the corresponding results based on the 24-hour bandwidth. Generally speaking, we find consistent effects across all attacks: the tweets posted 24 hours after the attacks convey more negative feelings than those posted 24 hours before the attacks. The differences in the magnitude of the estimates can be attributed to the context surrounding the attacks. For instance, the effects appear to be stronger and statistically more robust for the 2017 Manchester Arena bombing and the 2017 London Bridge attack, owing to the fact that these attacks had a high number of victims, widespread media coverage and Islamist perpetrators.

Table S.5: The emotional effect of terrorism: individual attacks

	Negative (1)	Fear (2)	Anger (3)	Sadness (4)	Disgust (5)
Panel A: 2016 Jo Cox Murder					
24-hour bandwidth	0.010***	0.009***	0.010***	0.004***	0.005***
	(0.001)	(0.001)	(0.001)	(0.001)	(0.001)
Observations	1,464,544	1,464,544	1,464,544	1,464,544	1,464,544
Panel B: 2017 Westminster Attack					
24-hour bandwidth	0.009**	0.018***	0.011***	0.004	0.007***
	(0.003)	(0.001)	(0.001)	(0.003)	(0.001)
Observations	912,502	912,502	912,502	912,502	912,502
Panel C: 2017 Manchester Arena Bombing					
24-hour bandwidth	0.024***	0.033***	0.021***	0.014***	0.018***
	(0.003)	(0.001)	(0.001)	(0.003)	(0.001)
Observations	907,344	907,344	907,344	907,344	907,344
Panel D: 2017 London Bridge Attack					
24-hour bandwidth	0.013***	0.025***	0.010***	0.008***	0.005***
	(0.001)	(0.001)	(0.001)	(0.001)	(0.001)
Observations	1,009,967	1,009,967	1,009,967	1,009,967	1,009,967
Panel E: 2017 Finsbury Park Attack					
24-hour bandwidth	0.002	0.003**	0.003*	-0.000	0.000
	(0.001)	(0.001)	(0.001)	(0.001)	(0.001)
Observations	828,258	828,258	828,258	828,258	828,258
Panel F: 2017 Parsons Green Bombing					
24-hour bandwidth	0.003**	0.004***	0.000	0.002**	0.001
	(0.001)	(0.001)	(0.001)	(0.001)	(0.001)
Observations	904,900	904,900	904,900	904,900	904,900
Panel G: 2019 London Bridge Stabbings					
24-hour bandwidth	0.007***	0.011***	0.006***	0.001	0.001
	(0.001)	(0.001)	(0.001)	(0.001)	(0.001)
Observations	809,494	809,494	809,494	809,494	809,494
Panel H: 2020 Reading Stabbings					
24-hour bandwidth	0.001	0.001	0.001	0.003**	-0.000
	(0.001)	(0.001)	(0.001)	(0.001)	(0.001)
Observations	806,093	806,093	806,093	806,093	806,093

Notes: The table shows the short-term effect of individual terrorist attacks on the respective sentiment or emotion. The short term effect is measured using the 24-hour bandwidth. Standard errors are clustered at the individual-level and reported in parentheses. * $p < .10$; ** $p < .05$; *** $p < .01$.

B.7 Heterogeneity analysis: time-to-event figures

In this section, we present the time-to-event figures for the attack groups we considered in our 'heterogeneity analysis' (see *Results* section): (i) the six attacks with Islamist perpetrators versus the two attacks with far-right perpetrators (Figure S.11); (ii) the four attacks with the highest number of victims versus the remaining four attacks (Figure S.12); and (iii) the four attacks with the highest media coverage versus the remaining four attacks (Figure S.13). For brevity and comparability, we focus on the three outcomes with the most pronounced post-attack effects: the overall negative sentiment, and the emotions of fear and anger. Overall, the patterns displayed in these figures support our key conclusions. First, attacks motivated by a radical interpretation of Islam result in more fearful sentiments than far-right attacks. Second, attacks with a high number of victims and extensive media attention elicit more negative sentiment and emotional responses than those with relatively fewer victims and less media coverage, and the corresponding effects last longer.

Figure S.11: Time-to-event analysis: far-right versus Islamic attacks

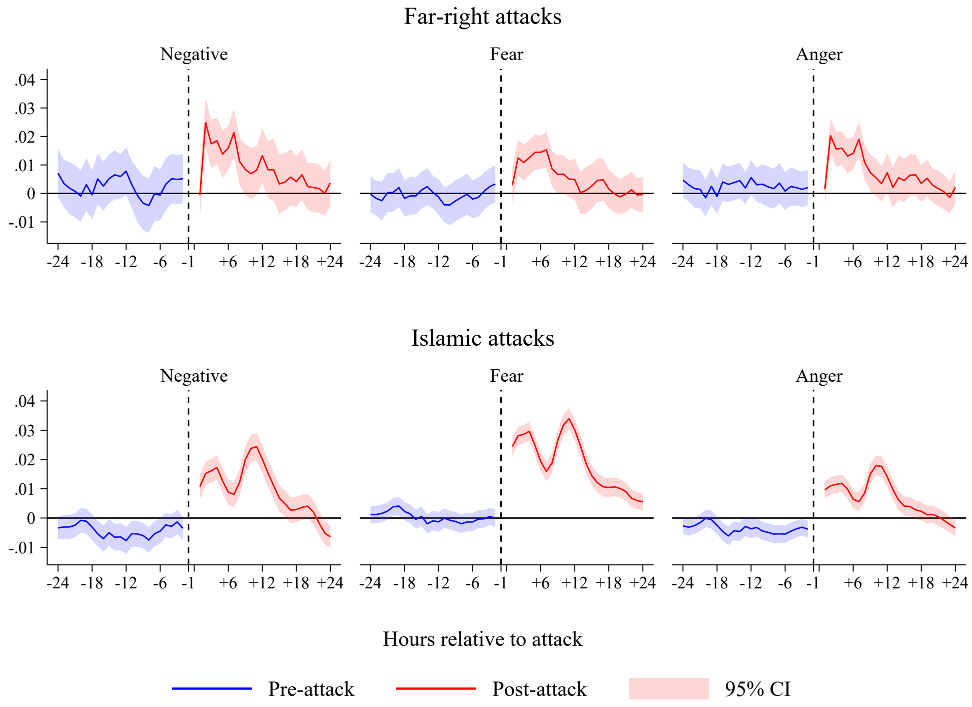

Notes: The figure shows the evolution of negative feelings 24 hours before and 24 hours after the sampled attacks. The tweets are aggregated at the hour level. The blue (red) solid line shows the 3-hour moving average estimates before (after) the attacks, taking the hour before the attack as the baseline. The tweets posted in the hour after the attack are dropped from the estimations. The shaded areas show the 95 percent confidence intervals.

Figure S.12: Time-to-event analysis: high-victim versus low-victim attacks

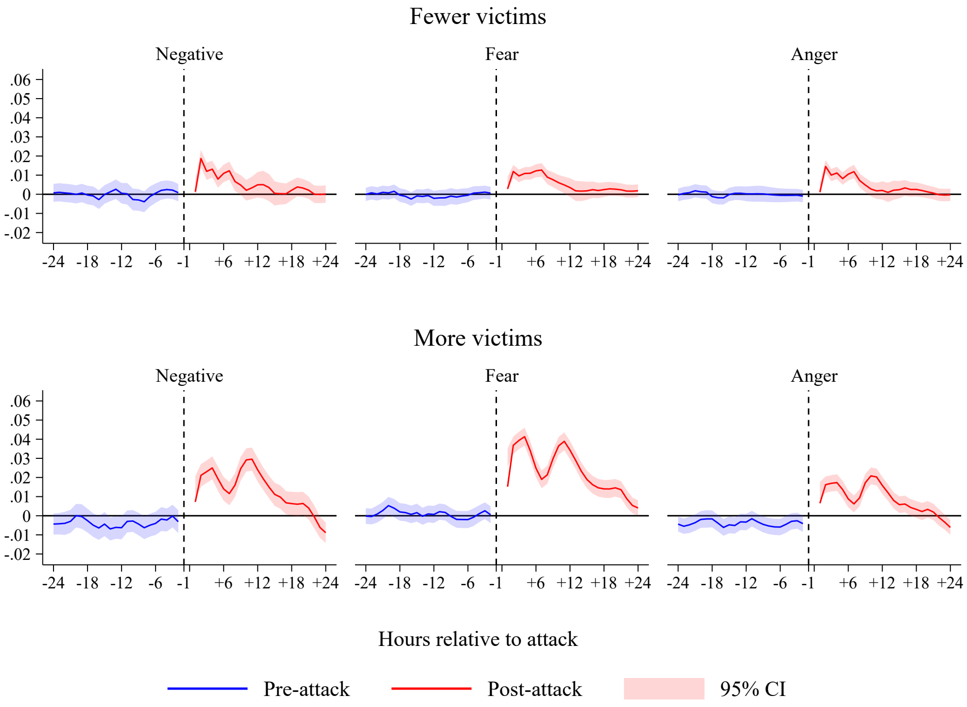

Notes: The figure shows the evolution of negative feelings 24 hours before and 24 hours after the sampled attacks. The tweets are aggregated at the hour level. The blue (red) solid line shows the 3-hour moving average estimates before (after) the attacks, taking the hour before the attack as the baseline. The tweets posted in the hour after the attack are dropped from the estimations. The shaded areas show the 95 percent confidence intervals.

Figure S.13: Time-to-event analysis: high-coverage versus low-coverage attacks

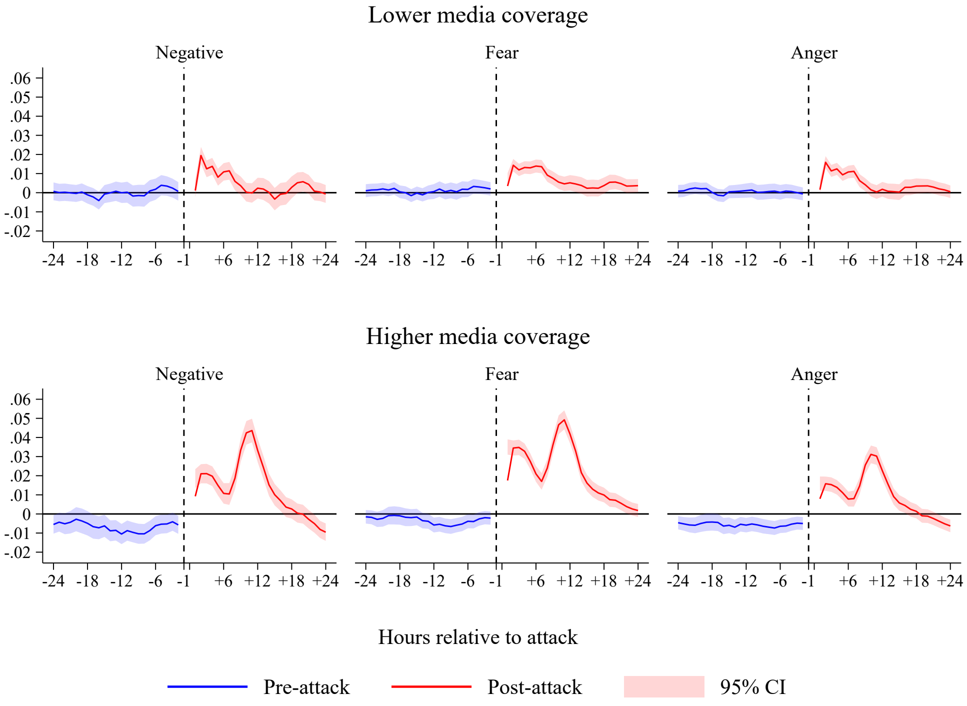

Notes: The figure shows the evolution of negative feelings 24 hours before and 24 hours after the sampled attacks. The tweets are aggregated at the hour level. The blue (red) solid line shows the 3-hour moving average estimates before (after) the attacks, taking the hour before the attack as the baseline. The tweets posted in the hour after the attack are dropped from the estimations. The shaded areas show the 95 percent confidence intervals.

C References

Bove, V., Efthyvoulou, G., and Pickard, H. (2022). Did terrorism affect voting in the Brexit referendum? *British Journal of Political Science*, 52(3):1133–1150.

Bove, V., Efthyvoulou, G., and Pickard, H. (2024). Are the effects of terrorism short-lived? *British Journal of Political Science*, 54(2):536–545.

Hutto, C. and Gilbert, E. (2014). Vader: A parsimonious rule-based model for sentiment analysis of social media text. In *Proceedings of the International AAAI Conference on Web and Social Media*, volume 8, pages 216–225.

Jones, H. (2020). More in common: the domestication of misogynist white supremacy and the assassination of Jo Cox. In *Racial Nationalisms*, pages 39–57. Routledge.

Loria, S. et al. (2018). textblob Documentation. *Release 0.15*, 2(8).

Mohammad, S. and Turney, P. (2010). Emotions evoked by common words and phrases: Using Mechanical Turk to create an emotion lexicon. In *Proceedings of the NAACL HLT 2010 Workshop on Computational Approaches to Analysis and Generation of Emotion in Text*, pages 26–34, Los Angeles, CA. Association for Computational Linguistics.

Mohammad, S. M. and Turney, P. D. (2013). Crowdsourcing a word-emotion association lexicon. *Computational Intelligence*, 29(3):436–465.

Muñoz, J., Falcó-Gimeno, A., and Hernández, E. (2020). Unexpected event during survey design: Promise and pitfalls for causal inference. *Political Analysis*, 28(2):186–206.

Nussio, E., Böhmelt, T., and Bove, V. (2021). Do terrorists get the attention they want? Comparing effects of terrorism across Europe. *Public Opinion Quarterly*, 85(3):900–912.

Pickard, H., Efthyvoulou, G., and Bove, V. (2023). What's left after right-wing extremism? the effects on political orientation. *European Journal of Political Research*, 62(1):338–351.

Zheng, X., Han, J., and Sun, A. (2018). A survey of location prediction on twitter. *IEEE Transactions on Knowledge and Data Engineering*, 30(9):1652–1671.
